# A humic acid loaded nano-zero-valent iron composite for enhanced immobilization of chromium and cadmium from soil

**DOI:** 10.3389/fpls.2026.1894863

**Published:** 2026-07-27

**Authors:** Xi Chen, Jianwei Yang, Lin He, Wenxia Wang, Xiangyu Xu, Muhammad Shaaban, Yajun Cai, Qi-an Peng

**Affiliations:** 1 School of Chemistry and Environmental Engineering, Wuhan Textile University, Wuhan, China; 2 Institute of Plant Protection and Soil Fertilizer, Hubei Academy of Agricultural Sciences, Wuhan, China; 3 College of Agriculture, Henan University of Science and Technology, Luoyang, China; 4 Clean Production of Textile Printing and Dyeing Engineering Research Center, Ministry of Education, Wuhan, China

**Keywords:** cadmium, chromium, immobilization, microbes, nano zero-valent iron, soil

## Abstract

**Introduction:**

As the global demand for soil pollution control becomes increasingly urgent, innovative material application strategies are essential for remediating soils co-contaminated with chromium (Cr) and cadmium (Cd) by efficiently immobilizing heavy metal ions and promoting their stabilization. This study evaluated humic acid-loaded nanoscale zero-valent iron (nZVI@HA) for the remediation of Cr- and Cd-contaminated soil.

**Methods:**

The remediation performance and underlying mechanisms of nZVI@HA were systematically investigated through soil incubation experiments, metal speciation analysis, high-throughput sequencing, and quantitative real-time PCR.

**Results:**

nZVI@HA achieved immobilization efficiencies of 58.19% for available Cr and 35.84% for available Cd. On day 50 of remediation, exchangeable Cr and Cd were markedly transformed into residual, Fe–Mn oxide-bound, and carbonate-bound fractions, substantially reducing their mobility and bioavailability. Concurrently, nZVI@HA alleviated Cr(VI) and Cd(II) stress, increased microbial community diversity, and improved soil fertility. The enrichment of taxa associated with ChrA, CzcA, and NitR suggests that nZVI@HA may enhance microbial resistance to Cr(VI) and Cd(II), potentially contributing to Cr and Cd immobilization.

**Discussion:**

The immobilization mechanisms primarily involved chemical reduction, adsorption, ion exchange, and microbially mediated processes. The synergistic effects between nZVI@HA and soil microorganisms make nZVI@HA an efficient and environmentally benign remediation material, providing an effective strategy for treating soils co-contaminated with heavy metals.

## 1 Introduction

As a 3d transition metal with redox activity, chromium primarily occurs in two oxidation states: trivalent Cr(III) and hexavalent Cr(VI). It finds extensive application in various industrial sectors, including electroplating, leather tanning, textile manufacturing, and dyeing processes (Daulton et al., 2020). Similarly, cadmium—recognized as a highly toxic heavy metal—is of considerable importance in electroplating, mining, and smelting industries (Wang et al., 2022; Kumar et al., 2022). The sources of chromium and cadmium contamination in the environment are mainly anthropogenic, primarily due to industrial discharges (Xia et al., 2019). Environmental pollution by Cr(VI) and Cd(II) poses significant risks to human health and ecosystems, with Cr(VI) causing various health problems, including skin rashes, gastric ulcers, respiratory issues, immune suppression, kidney and liver damage, and lung cancer (Jaiswal et al., 2018; Jiang et al., 2021). Exposure to Cd(II) is associated with kidney damage, bone fragility, lung damage and cancer (Oladipo et al., 2016). In plants, Cr and Cd inhibit shoot and root growth and affects nutrient uptake, homeostasis and physiological processes such as photosynthesis (Manel et al., 2016; Haider et al., 2024). Plants also face several detrimental effects associated with Cd and Cr stresses, including production of reactive oxygen species (ROS), disruption of hormonal balance, and damage to deoxyribonucleic acid (DNA), all of which cause stunted growth and even death of plants (Algethami et al., 2024). Therefore, the immobilization of heavy metals in soil is a critical prerequisite for improving the root-zone environment and reducing metal-induced toxicity and nutritional stress in plants.With the acceleration of industrialization, the combined pollution of Cr and Cd in farmland soil has become increasingly prominent, mainly manifested as decreased soil fertility, reduced soil enzyme activity and microbial activity, and inhibition of crop growth, thereby posing food safety risks and threatening human health. Therefore, there is an urgent need to find an efficient, safe and economical method for Cr,Cd-contaminated soil remediation.

In recent years, the application of nanotechnology and nanomaterials has demonstrated considerable promise for soil remediation (Gopinath et al., 2021). The application of nanoscale zero-valent iron (nZVI) for *in-situ* remediation of soils contaminated with Cr(VI) and Cd(II) has garnered significant attention (Jin et al., 2023a). With its high surface area and strong reducing capability, nZVI effectively reduces toxic Cr(VI) to less toxic Cr(III), thereby lowering its bioavailability and toxicity in soil (Chen et al., 2020).For instance, Yang et al. successfully synthesized a nano zero-valent iron composite material (nZVI@TP-Mont),which showed excellent Cr(VI) removal (almost complete removal) in soil and water at pH 2.2, while 84% removal was achieved at pH 6.0 (Yang et al., 2021). Peipei et al. remediated cadmium-contaminated soil by loading nano-zero-valent iron with biochar, and the results showed that nZVI@BC exhibited superior Cd immobilization effects,and the abundance, diversity, and species composition of soil microbial communities were altered by nZVI@BC (Peipei et al., 2024). Guo et al. investigated the effectiveness and mechanism of sulfidated nanoscale zero-valent iron (S-nZVI) for Cd-contaminated soil remediation, finding that the exchangeable (EX) Cd was reduced by more than 97.6% at the optimal dosage of 5 g/kg (Guo et al., 2021). In addition, Ren et al. demonstrated that the use of palagonite-coated nano zero-valent iron for stabilizing cadmium in contaminated soil effectively reduced the bioavailability and mobility of Cd in the soil (Ren et al., 2020). Jin et al. applied BC-BE-nZVI as a soil amendment to systematically investigate its remediation effects on Cd, Cr, and Pb contamination (Jin et al., 2023b). BC-BE-nZVI could effectively immobilize Cd, Cr, and Pb in soil and significantly improve the transformation of Cd, Cr, and Pb fractions to less bioavailable fractions, thereby decreasing their mobility. Beyond metal stabilization, the remediation process enhanced the soil microbial community, as evidenced by increased bacterial diversity, richness, and enzyme activities. In addition, the application of nZVI can be a useful strategy to immobilize As, Cr, Pb and Zn in calcareous or acidic soils in both single- or multi-metal(loid) contamination conditions (Gil-Díaz et al., 2017). Overall, nano zero-valent iron and its composite materials have shown promising promising potential for immobilizing Cr(VI) and Cd(II) in soil (Chen et al., 2017).

Humic acid (HA) is a large molecule that constitutes humus and has a porous and complex structure (Francioso et al., 2019). It is widely present in soil, water, and sediments, and its abundant functional groups, such as carboxyl and phenolic hydroxyl groups, can enhance soil fertility and promote pollutant removal (Zhu et al., 2023). Humic acid is recognized as a valuable soil amendment in phytoremediation practices due to its ability to enhance microbial diversity, stimulate enzymatic activities such as urease and sucrase, and support early plant development under heavy metal stress. Some studies have shown that HA can significantly reduce the toxicity of nZVI to indigenous microorganisms (Hou et al., 2019). The organic nature of HA enables it to exert pronounced positive effects on plant growth, reduce heavy metal toxicity, and improve phytoremediation performance in polluted soils through the attenuation of oxidative stress (Canal et al., 2022; Boysan Canal, 2025). Previous work showed that the co-application of HA and nZVI could synergistically enhance Cr(VI) removal from contaminated soil and reduce Cr accumulation in plants (Sun et al., 2020). Loading nZVI onto HA can enhance its dispersibility, stability, and electron transfer ability, effectively inhibiting aggregation and passivation (Yao et al., 2019), making nZVI@HA an efficient and environmentally friendly environmental remediation material (Fu et al., 2016).

While several recent reports have confirmed the efficacy of nZVI@HA for the remediation of individual Cr or Cd contamination, its behavior, efficiency, and ecological impact in soils burdened with the coexistence of multiple heavy metals remain underexplored.Only a few studies have addressed the simultaneous immobilization of multiple heavy metals in soil due to the complex interactions among metals (e.g., synergistic, additive, or antagonistic effects). Furthermore, limited information is available on how modified nZVI influences soil microbial community composition and enzyme activities.Based on the above context, the primary innovation of this paper lies in bridging the gap between remediation efficacy in multi-metal contaminated soils and comprehensive soil ecological safety assessments. Therefore, this paper investigates the immobilization performance and mechanisms of Cr(VI) and Cd(II) in soil by nZVI@HA composites, and further explores the effects of nZVI@HA on soil enzyme activity and the diversity and community structure of indigenous microorganisms, with the aim of providing a theoretical basis for the green remediation of heavy metal contaminated soil.

## 2 Materials and methods

### 2.1 Preparation of nZVI@HA

Based on the method of Liu et al. and considering both economic viability and the removal efficiency of Humic Acid Loaded Nano-Zero-Valent Iron(nZVI@HA)for Cr(VI) and Cd(II), an HA: Fe(II) mass ratio of 3:1 was selected to synthesize the nZVI@HA used in the subsequent soil experiments (Liu et al., 2025).The specific preparation method can be found in Text S1.

### 2.2 Soil material

The test soil used for the artificial simulation of contaminated soil in agricultural land was taken from farmland in Zhongjing Village, Geji River Basin, Dudao District, Jingmen City, Hubei Province (E: 112.2633492, N: 30.95923452). Main soil properties can be found in **Supplementary Table 1** and **Text S2**. After the aging treatment, as described in Text S3, the pollution status was determined as follows: The total chromium content in the single chromium-contaminated soil was 496.54 mg/kg. The total cadmium content in the single cadmium-contaminated soil was 2.47 mg/kg, and the total chromium content was 495.43 mg/kg and the total cadmium content was 2.51 mg/kg in the Cr, Cd-contaminated soil.

### 2.3 Soil culture test

This experiment evaluated the remediation effects of 10 different material treatments on different contaminated soils, as detailed in **Table 1**. Each treatment had three replicates. For Cr-contaminated soil, Cd-contaminated soil, and Cr,Cd-contaminated soil, 200 g of soil and the corresponding material at a rate of 10 g/kg were weighed into small pots (200 mL capacity) and thoroughly mixed. Then, 70 mL of deionized water was added to each pot, and the soil moisture content was maintained at 35% throughout the experiment. The pots were covered with perforated plastic wrap and incubated in a constant-temperature incubator at 25 ± 0.2°C. On days 1, 30, and 50, 15 g of moist soil was sampled from each pot for analysis.

**Table 1:** Method of treatments.

Numbering	Method of handling
CK	There are no additives in the soil
S1	Remediation of Cr-contaminated soil by HA
S2	Remediation of Cd-contaminated soil by HA
S3	Remediation of Cr, Cd-contaminated soil by HA
S4	Remediation of Cr-contaminated soil by nZVI
S5	Remediation of Cd-contaminated soil by nZVI
S6	Remediation of Cr, Cd-contaminated soil by nZVI
S7	nZVI@HA remediation of Cr-contaminatedsoil
S8	nZVI@HA remediation of Cd-contaminated soil
S9	nZVI@HA remediation of Cr, Cd-contaminated soil

### 2.4 Analysis of soil properties

To analyze the changes in soil properties during the remediation process, soil pH, cation exchange capacity (CEC), dissolved organic carbon (DOC), and available cadmium and chromium concentrations were examined on days 1, 30, and 50 of the remediation period. The detailed analysis of soil properties is presented in Text S4.

### 2.5 Analysis of soil enzyme activity

To investigate the impact of soil remediation materials on microbial activity in soil, variations in catalase, urease, and sucrase activities were examined before and after different treatment processes.The specific method for analyzing soil enzyme activity is described in Text S5.

### 2.6 Analysis of soil microbial community structure

To explore the effect of the addition of nZVI@HA on the microbial community structure of different contaminated soils, this study conducted soil microbial community analyses on four treatment groups: CK, S7, S8 and S9. All soil samples were cryopreserved at -4°C and sent to Personalbio for 16S rRNA gene diversity detection, including species composition, alpha diversity, beta diversity, ASV/OTU Venn diagrams and species composition heat map analysis. In addition, to assess the effect of nZVI@HA on the expression of chromium and cadmium-related genes, the absolute expression levels of Cr and Cd-related genes in the four groups of CK, S7, S8 and S9 were measured by qPCR fluorescence quantification. The 16S_V3-V4a gene sequence method was used to analyze the diversity of soil microbial communities.

### 2.7 Statistical analysis

All statistical analyses were performed using SPSS software. Each treatment was conducted with three independent replicates (n=3). Statistical significance was considered at p<0.05. One-way analysis of variance and multiple comparisons (Duncan’s method) were performed. Data processing and graphing were conducted using Origin 2024.

## 3 Results and Discussion

### 3.1 Characterization of nZVI@HA

**Figure 1:**
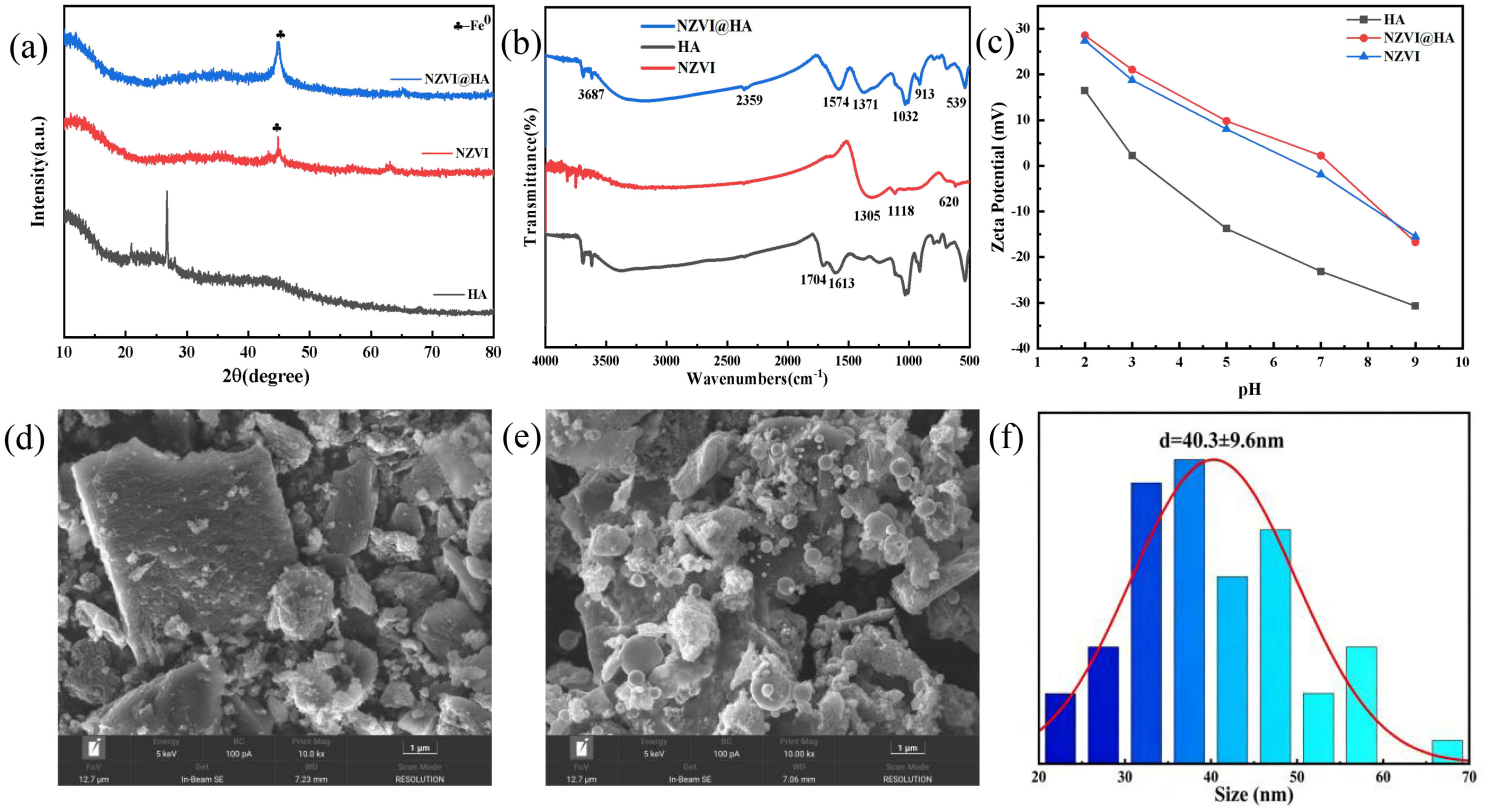
XRD spectra of HA, nZVI, and nZVI@HA **(a)**, FTIR spectra of nZVI@HA,HA and nZVI **(b)**, Zeta potential of HA, nZVI, and nZVI@HAat different pH levels **(c)**. Scanning Electron Microscopy (SEM) images of **(d)** Humic Acid (HA) and **(e)** nZVI coated with HumicAcid (nZVI@HA), along with the corresponding particle size distribution **(f)**.

X-ray diffraction (XRD) analysis (**Figure 1a**) revealed that HA exhibited an amorphous carbon diffraction peak at 2θ=26.13°, while nZVI and nZVI@HA both showed a Fe^0^ diffraction peak at 44.9°. The disappearance of the characteristic HA peak is primarily attributed to the “masking effect” from the strong signal of highly crystalline nZVI and the disruption of the HA stacking structure caused by the *in-situ* growth of nanoparticles, which provides compelling evidence for the successful loading of nZVI and the strong interfacial interaction between the two components (Dong et al., 2017).

As observed in the FTIR spectra (**Figure 1b**), both nZVI and nZVI@HA exhibited distinct characteristic iron peaks compared with pure HA. Specifically, the peaks at 539 cm^-1^, 3687 cm^-1^, and 689 cm^-1^ correspond to the stretching vibrations of Fe-O, Fe-OH, and Fe-OOH, respectively. Furthermore, the characteristic groups originally present in HA, such as carboxyl (COOH), hydroxyl (OH), aldehyde (CHO), and carbon-carbon triple bonds (C≡C), were not observed in the nZVI@HA composite. This absence is likely attributable to the physical masking of the HA functional groups by the loaded nZVI nanoparticles (Xu et al., 2019). **Figure 1c** shows the zeta potentials of HA, nZVI, and nZVI@HA at pH values of 2, 3, 5, 7, and 9.

As shown in **Figure 1d-f**, SEM imaging confirmed that nZVI was successfully loaded onto the HA surface; the resulting nZVI@HA composite exhibited a distinctly rough and uneven morphology, with the well-dispersed iron particles being predominantly under 70 nm in size.

In **Supplementary Figure S1**, XPS survey of nZVI@HA detected C1s (285.36eV, 39.26%), O1s (531.37eV, 50.64%), and Fe2p (711.34eV, 10.10%). High-resolution C1s was resolved into C-C (284.87eV, 64.87%), C-O (286.15eV, 34.36%), and C=O (288.67eV, 0.77%), while O1s exhibited Fe-O (531.23eV, 89.44%) and C-O (529.53eV, 10.56%). In the Fe2p fitting analysis, Fe(III) (Fe2p_1/2_ at 725.56 eV; Fe2p_3/2_ at 712.62eV), Fe(II) Fe2p_1/2_ at 723.58eV; Fe2p_3/2_ at 710.47eV), andFe^0^(718.09eV) were successfully identified. Concurrently, the emergence of the Fe-O peak indicates surface oxidation of nZVI@HA, aligning with typical nZVI-based composites (Chen et al., 2023; Khalil et al., 2018; Wang et al., 2018).

Collectively, the XPS, XRD, FTIR, and SEM results confirmed the successful loading of nZVI onto HA.

### 3.2 Effects of nZVI@HA on morphological changes of available Cr and Cd in Cr, Cd-contaminated soil

#### 3.2.1 The effect of nZVI@HA on the removal of available Cr and Cd in contaminated soil

**Figure 2:**
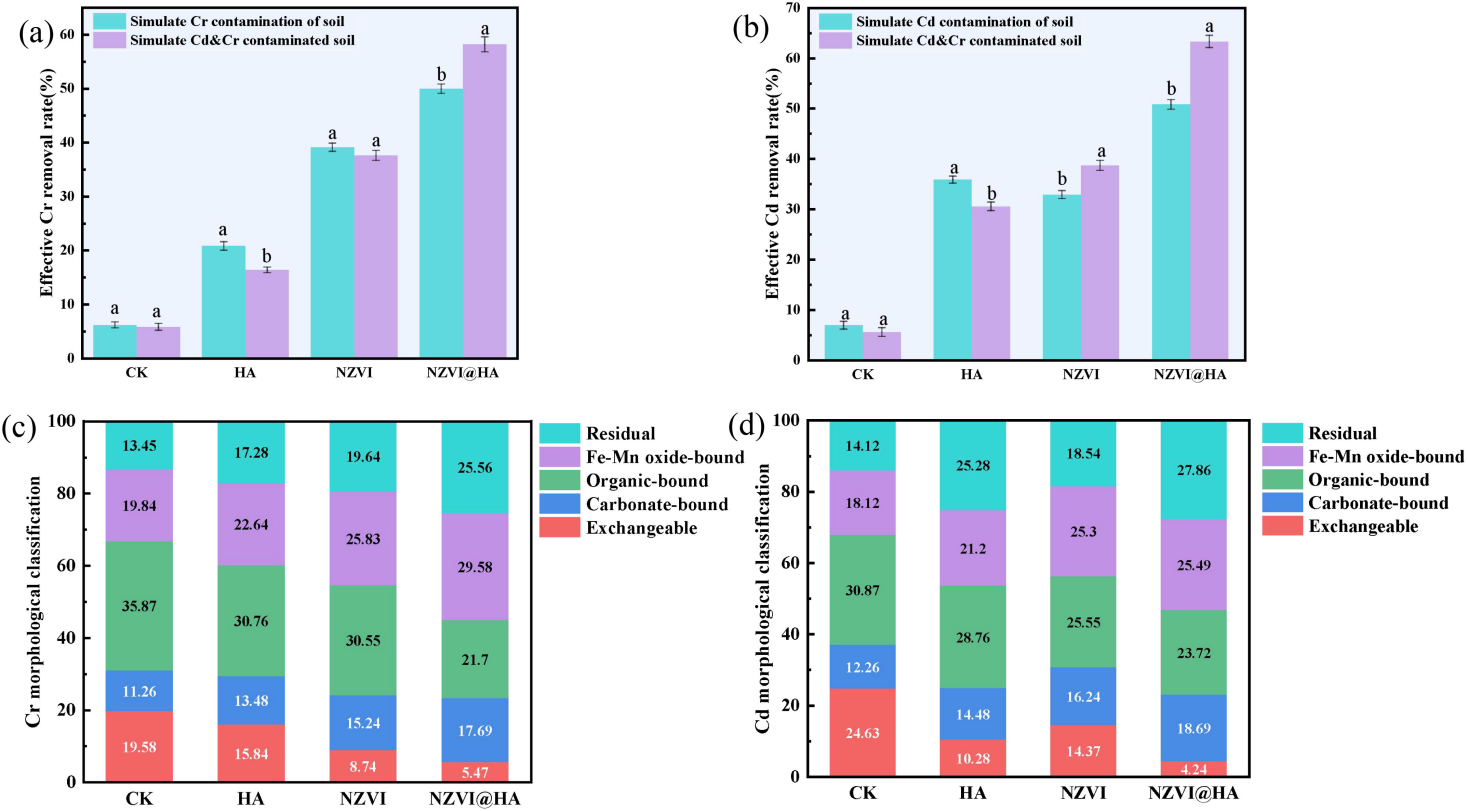
The removal effect of different materials on the available Cr in Cr-contaminated soil, Cr, Cd-contaminated soil **(a)**, the removal effect of different materials on the available Cd in Cd-contaminated soil, Cr, Cd-contaminated soil **(b)** (Data are presented as mean ± SD (n = 3). Different lowercase letters indicate significant differences among treatments according to Duncan’s multiple range test at p < 0.05.), Fractionation of Cr **(c)** and Cd **(d)** species during the removal of Cr and Cd contamination by different materials.

The available Cr and Cd concentrations in the soil can be detected by the diethylenetriaminepentaacetic acid extraction method. **Figure 2a** indicates that the addition of HA, nZVI, and nZVI@HA each enhanced the removal efficiency of available Cr from the contaminated soil. Among them, nZVI@HA had a higher removal rate of available Cr in Cr, Cd-contaminated soil (58.19%) than in Cr-contaminated soil (49.97%). The removal rate of available Cr in Cr,Cd-contaminated soil by HA (16.42%) was lower than that in Cr-contaminated soil (20.84%), possibly due to the limited adsorption or complexation capacity of HA in Cr,Cd-contaminated soil (Jingzi et al., 2018), nZVI showed no significant difference (p >0.05) in the removal of available Cr from Cr, Cd-contaminated soil and Cr-contaminated soil.

**Figure 2b** reveals a difference in HA’s performance, showing a higher available Cd removal rate in the Cd-contaminated soil (35.84%) than in the Cr,Cd-contaminated soil (30.53%). In contrast, nZVI and nZVI@HA had higher removal rates of available Cd in Cr,Cd-contaminated soil than in Cd-contaminated soil. This phenomenon may be attributed to the presence of nZVI. During the partial reduction of Cr(VI) to Cr(III), oxidation products such as Fe(II) and Fe(III) are generated, which can subsequently form insoluble complexes or precipitates with Cd(II). This process facilitates the immobilization of Cd(II) within the soil matrix (Liang et al., 2022).

This difference may be related to changes in the soil chemical environment caused by the coexistence of Cd, which could influence the interaction between nZVI@HA and Cr species. Previous studies have suggested that coexisting metal ions may affect electron-transfer processes during remediation (Liang et al., 2022).

#### 3.2.2 The speciation changes in Cr,Cd-contaminated soil

**Figure 2c** and **Figure 2d** present the speciation fractionation of Cr,Cd-contaminated soil using the Tessier five-step sequential extraction procedure, illustrating the distribution among residual, Fe-Mn oxide-bound, organic-bound, carbonate-bound, and exchangeable across different materials.

As shown in **Figures 2c, d**, after the addition of HA, nZVI, and nZVI@HA, the exchangeable fraction of Cr decreased from 19.58% to 15.84%, 8.74%, and 5.47%, respectively, compared with CK.The corresponding exchangeable Cr concentration decreased from approximately 195.8 mg kg^-^¹ to 158.4, 87.4, and 54.7 mg kg^-^¹, representing absolute reductions of 37.4, 108.4, and 141.1 mg kg^-^¹, respectively. Similarly, the exchangeable fraction of Cd decreased from 24.63% to 10.28%, 14.37%, and 4.24% respectively, the exchangeable Cd concentration decreased from approximately 0.985 mg kg^-^¹ to 0.411, 0.575, and 0.170 mg kg^-^¹, corresponding to absolute reductions of 0.574, 0.410, and 0.816 mg kg^-^¹, respectively.

The results can be attributed to the combined effects of nZVI’s strong reducibility and HA’s adsorption capacity, potentially providing greater surface area and more active sites (Dai et al., 2022). nZVI@HA helps to transform Cr and Cd from the exchangeable state into more stable residual and carbonate-bound states, reducing their mobility and bioavailability in the soil environment.The iron-manganese oxide fraction and carbonate-bound fraction exhibited varying degrees of increase. With nZVI@HA as a strong reducing agent, Fe^0^ may be oxidized to Fe(III) when reacting with Cr(VI), thereby increasing the formation of iron oxides, which help adsorb and stabilize heavy metals Cr and Cd in the soil, thus increasing the content of Fe-Mn oxide-bound (Zhang et al., 2023).Reactive iron phases may contribute substantially to contaminant retention and stabilization through adsorption and co-precipitation processes in soils (Silva et al., 2024). In addition, the addition of nZVI@HA may have altered the activity of carbonate ions in the soil, promoting the complexation of heavy metals with carbonate, resulting in an increase in the carbonate-bound (Yu et al., 2020).

### 3.3 The effects of nZVI@HA on soil properties

Alterations in soil physicochemical properties may substantially influence nutrient availability and biological activity, thereby affecting overall soil functioning and ecosystem recovery processes (Yuan et al., 2024). The efficiency of immobilization of Cr and Cd by nZVI@HA soil by nZVI@HA is governed by three key parameters: pH, cation exchange capacity (CEC), and dissolved organic carbon (DOC) levels.Data on soil pH, CEC, and DOC after 50 days of remediation with different treatments are shown in **Table 2**.

**Table 2:** Properties in the different treatments assayed.

Soil properties	Time	Ck	S1	S4	S7	S2	S5	S8	S3	S6	S9
pH	Day 1	6.28	6.25	6.5	6.32	6.43	6.48	6.39	6.38	6.22	6.54
	Day 50	6.32	6.74	6.62	6.49	6.35	6.47	6.35	6.44	6.58	6.94
CEC	Day 1	240.87	239.04	241.06	235.98	211.35	218.07	225.41	242.06	245.17	246.35
(cmol^+^/kg)	Day 50	238.85	221.24	206.876	210.86	190.56	188.86	201.09	224.69	228.94	230.87
DOC	Day 1	200.09	199.87	198.99	201.96	200.78	206.47	203.36	208.69	219.63	213.84
(mg/kg)	Day 50	204.86	204.93	206.41	207.41	202.56	206.31	204.47	209.99	215.37	213.48

**Table 2** shows that after 50 days of remediation with the addition of HA, nZVI and nZVI@HA, the pH of Cr-contaminated soil and Cr, Cd-contaminated soil decreased slightly because nZVI may generate H^+^ during the reduction of Cr(VI) to Cr(III), which leads to a decrease in soil pH. The pH of the Cd-contaminated soil increased slightly, possibly due to the release of alkaline substances during the co-precipitation reaction of nZVI@HA with Cd, which may have contributed to a slight increase in pH.

The CEC of soil refers to its ability to adsorb and retain cations, and soil with a high CEC can adsorb heavy metal ions more effectively (Kalubi et al., 2017). Overall, after 50 days of remediation with HA, nZVI and nZVI@HA, the CEC of Cr-contaminated soil, Cd-contaminated soil and Cr, Cd-contaminated soil all decreased to some extent. This might be due to the reduction in pH values, which possibly decreased the number of negative charges in the soil and thus lowered the CEC (Shi et al., 2023). After 50 days of treatment with nZVI@HA on Cr-contaminated soil, Cd-contaminated soil and Cr,Cd-contaminated soil, the CEC decreased by 10.64%, 10.79% and 6.28% respectively. In addition, the occupation or blockage of exchange sites by Fe corrosion products, HA–metal complexes, or newly formed mineral precipitates may reduce the accessible exchange capacity of the soil (Gholami and Rahimi, 2023).In the long term, decreased CEC may weaken the soil’s buffering capacity for cationic nutrients and metals.

Dissolved organic carbon (DOC) can form complexes with heavy metals, affecting their migration and bioavailability in the soil (Liu et al., 2018). As shown in **Table 2**, after 50 days of remediation with the addition of HA, nZVI and nZVI@HA, the DOC in Cr-contaminated soil, Cd-contaminated soil and Cr, Cd-contaminated soil increased to some extent, which may be due to the materials altering the structure and activity of the soil microbial community, promoting microbial activity for decomposing organic matter and increasing the production of DOC (Gómez-Sagasti et al., 2018).The interaction between soil organic constituents and reactive surfaces plays a central role in regulating chemical retention, mobility, and bioavailability within agricultural soils (Sarkis et al., 2024). Increased DOC may promote metal immobilization by forming stable organo-metal complexes or facilitating adsorption onto reactive Fe mineral surfaces.

### 3.4 The effect of nZVI@HA on soil enzyme activity

**Figure 3:**
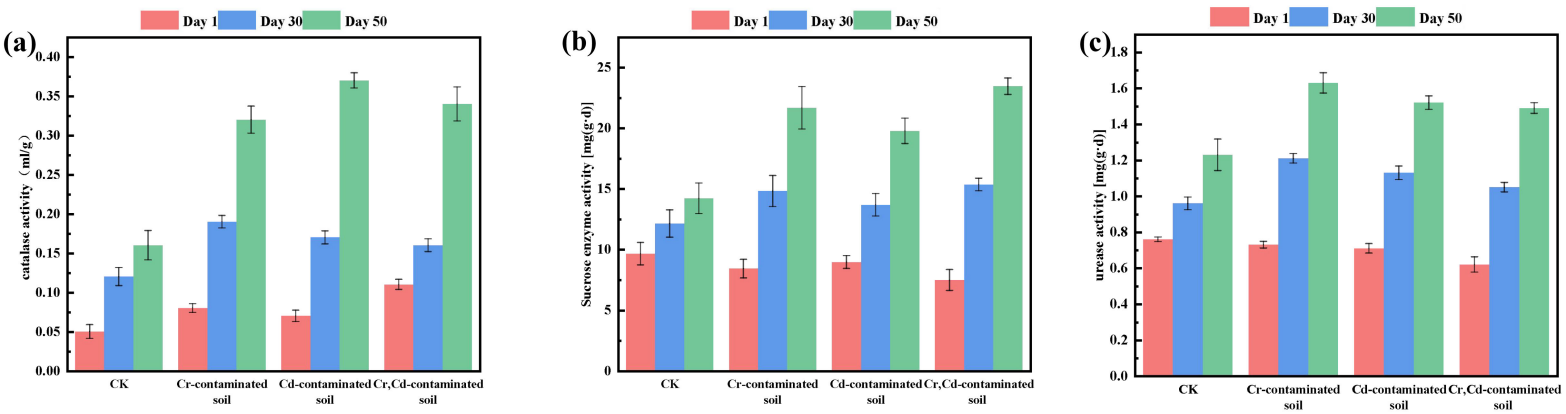
Effect of nZVI@HA on the activities of catalase **(a)**, sucrase **(b)** and urease **(c)** in the treatment of Cr-contaminated soil, Cd-contaminated soil and Cr, Cd-contaminated soil.

Catalase, urease, and sucrase can reflect the microbial activity and organic matter decomposition capacity of soil, which is crucial for exploring the interaction between nZVI@HA and the soil environment (Wang et al., 2021). The effects of nZVI@HA on catalase activity, sucrase activity, and urease activity in Cr-contaminated soil, Cd-contaminated soil, and the treatment of Cr,Cd-contaminated soil are shown in **Figure 3**.

The application of nZVI@HA consistently promoted catalase activity in Cr-, Cd-, and Cr,Cd-contaminated soils, with levels reaching 0.32, 0.37, and 0.34 mL/g, respectively (**Figure 3a**). This observation can be attributed to two potential factors: nZVI@HA likely mitigated the toxicity of Cr and Cd,and the increased catalase activity may signal a general improvement in overall soil biological activity (Liron et al., 2022).The impact of nZVI@HA on sucrase activity evolved over time (**Figure 3b**). On the first day, activities in all treated soils were slightly lower than in the CK, likely because nZVI@HA addition triggered resource competition, temporarily impairing microbial metabolism (Yang et al., 2023). In contrast, on day 50, sucrase activity increased substantially to 21.68, 19.78, and 23.46 mg/(g·d) in the respective soils.As shown in **Figure 3c**, after the addition of nZVI@HA, urease activity was the highest in Cr-contaminated soil (1.63mg/(g·d)), followed by urease activity in Cd-contaminated soil (1.52mg/(g·d)), and the lowest in Cr, Cd-contaminated soil (1.49mg/(g·d)). The increase in urease activity indicates that the nitrogen cycling function of the soil has been restored or enhanced, which is beneficial for improving soil fertility and promoting plant growth.Changes in soil physicochemical conditions induced by amendments may substantially modify microbial activity and ecosystem functioning, resulting in long-term alterations in soil biological processes (Lopes et al., 2024).

### 3.5 nZVI@HA effects on soil microbial communities

As novel modified iron-based soil conditioners, HA, nZVI and nZVI@HA can influence the structure of soil microbial communities, altered the microbial community composition, enhance the activity of related microorganisms. Analyses on the species composition and diversity of soil microbial communities may reveal the remediation mechanism of these materials on Cr, Cd-contaminated soils.

#### 3.5.1 Microbial community composition analysis

**Figure 4:**
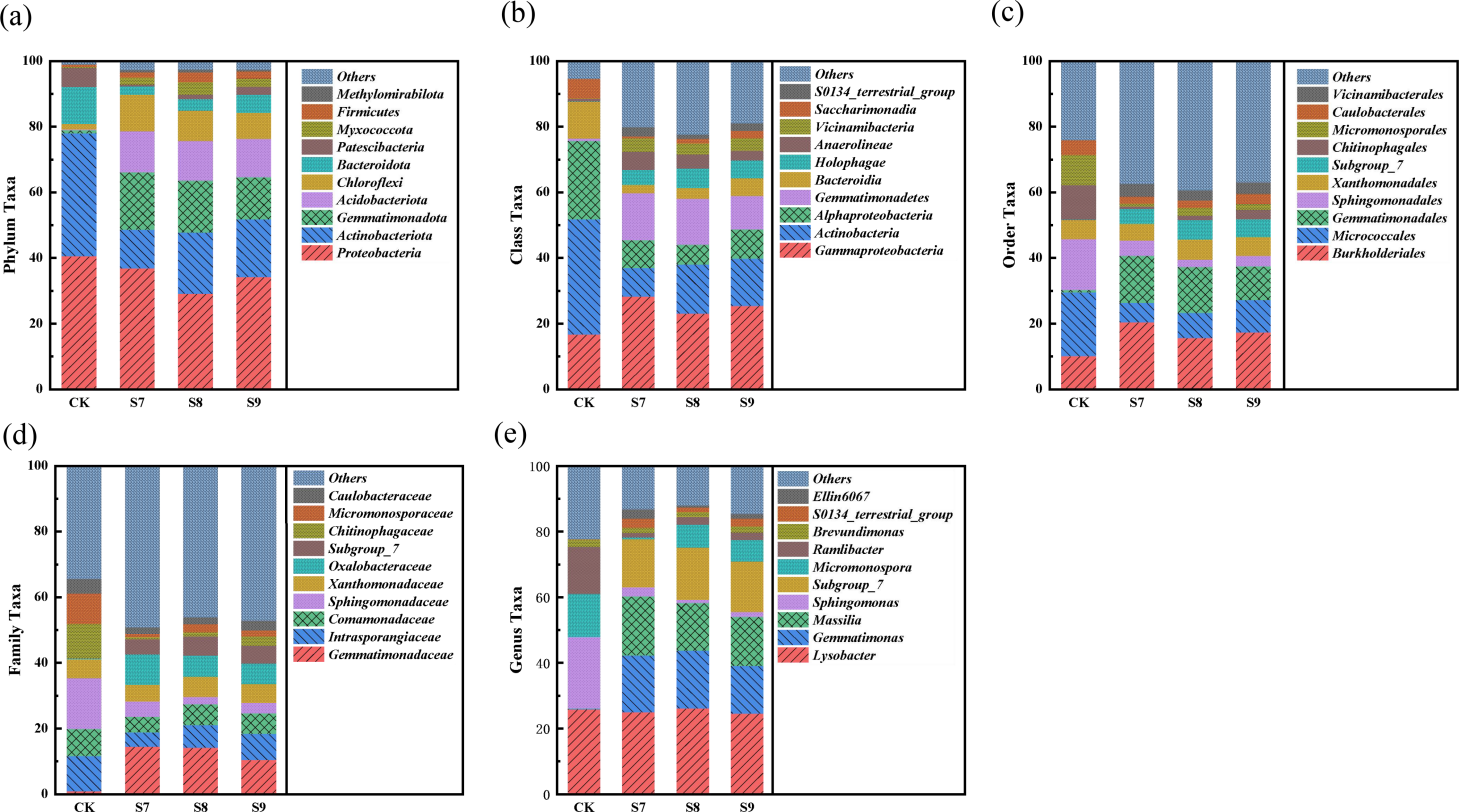
Relative abundance of microbial communities in soil at the phylum **(a)**, class **(b)**, order **(c)**, family **(d)** and genus **(e)** levels (CK: soil without any additives, S7: nZVI@ HA remediates Cr-contaminated soil, S8: nZVI@HA remediates Cd-contaminated soil, S9: nZVI@HA remediates Cr, Cd-contaminated soil).

Microbial community composition under nZVI@HA treatment was analyzed by constructing a feature table with singletons removed. **Figure 4** displays the relative abundance profiles for communities across all five taxonomic levels, revealing the structural differences among the soil treatments.

The relative abundance profiles of microbial communities at the phylum level are compared across the four treatments (CK, S7, S8, and S9) in **Figure 4a**. Proteobacteria, *Actinobacteriota*, *Chloroflexi*, and *Bacteroidota* were the dominant phyla (relative abundance greater than 1%), all of which were dominant phyla in chromium - and cadmium-contaminated soils. Among them, Proteobacteria was the dominant phylum, with relative fractions of 40.36%, 36.66%, 28.97% and 34.06% respectively.Compared with CK, S7, S8 and S9 showed increased abundances of *Gemmatimonadota*, *Acidobacteriota*, *Myxococcota* and *Firmicutes*, indicating that nZVI@HA as a soil remediation agent that influence the structure of the soil microbial community, improve the growth of related microorganisms, and increase the activity (Yan et al., 2021). Compared with S8, the relative abundance of dominant phyla in S9 was higher than that in S8. In summary, the microbial community composition at the phylum level increased, further demonstrating that the remediation material nZVI@HA promoted the growth of the microbial community.

**Figure 4b** displays the relative abundance profiles of the microbial community at the class level. In the four groups of treatments, CK, S7, S8 and S9, *Gammaproteobacteria*, *Actinobacteria*, *Alphaproteobacteria* and *Bacteroidia* were the dominant classes (relative abundance greater than 1%). All are dominant in chromium and cadmium-contaminated soils, with *Actinobacteria* being the dominant class in CK, with a relative fraction of 35.20%, and *Gammaproteobacteria* being the dominant class in S7, S8, and S9. The relative abundances were 28.51%, 22.92% and 25.23% respectively. Compared with CK, S7, S8 and S9 showed increased abundance of four dominant bacteria, namely *Gemmatimonadetes*, *Holophagae*, *Anaerolineae* and *Vicinamibacteria*, which further proved that nZVI@HA promotes microbial growth. However, it is worth noting that the relative abundance of *Actinobacteria* in the S7, S8 and S9 treatments was 8.65%, 14.90% and 14.47% respectively, which was much lower than that in the CK group. This is because the *Actinobacteria phylum* promotes the oxidation of the remediation agent nZVI@HA, resulting in iron precipitates (Tingting et al., 2022), which led to the formation of a passive layer on the surface of nZVI@HA, thereby inhibiting the soil remediation efficiency of nZVI@HA.

**Figure 4c** shows the relative abundance changes of the microbial community at the “order” level, further revealing the effects of nZVI@HA on the microbial community in combination with phylum level and class level. It can be seen from the figure that *Burkholderiales*, *Micrococcales*, *Sphingomonadales* and *Xanthomonadales* are the dominant orders (relative abundance greater than 1%). Among them, *Micrococcales* was the dominant order of CK, with a relative fraction of 9.96%, and *Burkholderiales* was the secondary dominant order of CK, with a relative fraction of 19.44%. In contrast, *Burkholderiales* were the dominant order of S7, S8 and S9, with relative abundances of 20.19%, 15.47% and 17.13% respectively, and *Micrococcales* were the secondary dominant order of S7, S8 and S9. The relative abundances were 6.02%, 7.66% and 9.94% respectively. Compared with CK, the newly added dominant orders in S7, S8 and S9 were *Gemmatimonadales* and *Subgroup_7*, and the reduced dominant orders were *Chitinophagales* and *Micromonosporales*, all of which were newly discovered microorganisms. Among them, *Gemmatimonadales* and *Subgroup_7* are Cr(VI)- and Cd(II)-tolerant bacteria.

Analysis of the microbial community composition at the family level (**Figure 4d**) revealed a significant increase in the relative abundance of *Gemmatimonadaceae* in S7, S8, and S9 compared to the CK group. This observation is in line with the findings of Jia et al (Jia et al., 2022). The relative abundance of the *Gemmatimonadaceae* population was significantly higher than that of the control soil. The dominant family of CK was *Sphingomonadaceae* with a relative abundance of 15.46%, and the secondary dominant families were *Intrasporangiaceae* and *Chitinophagaceae*. The relative abundances were 10.70% and 10.52 respectively. The dominant family for S7, S8 and S9 was the newly added *Gemmatimonadaceae*, with relative abundances of 14.31%, 13.99% and 10.20% respectively, and the secondary dominant family was *Oxalobacteraceae*, with relative abundances of 9.28%, 6.39% and 6.31% respectively.

The dynamics of microbial community composition at the genus level are illustrated in **Figure 4e**. The dominant genera in the S7, S8, and S9 groups were *Lysobacter*, *Gemmatimonas*, *Massilia*, *Subgroup_7*, *Sphingomonas*, and *Micromonospora*, *RamLibacter* and *S0134_terrestrial*_group. The dominant genera in the CK group were *Lysobacter* (25.64%) and *Sphingomonas*. 21.75%, *Micromonospora* (13.19%), and *RamLibacter* (14.33%). The number of dominant genera doubled in the S7, S8, and S9 groups compared to the CK group, and the newly added *Lysobacter* was the dominant genus in the samples of the S7, S8 and S9 groups, with relative abundance of 24.78%, 25.99% and 24.40%, respectively. The addition of *Gemmatimonas* and *Massilia*, which were also subdominant genera, further demonstrated that the addition of nZVI@HA promoted soil microbial diversity.

In summary, the presence of nZVI@HA enriched the composition and diversity of the soil microbial community structure, suggesting that nZVI@HA can improve the soil environment, reduced the bioavailable proportions, and was accompanied by changes in soil microbial community composition and the relative abundance of functional genes. Changes in soil environmental conditions can substantially restructure microbial communities and modify ecological interactions governing soil functioning (Cezar et al., 2025).For example, *Gemmatimonadota* may affect the dispersibility and activity of nZVI@HA by altering the organic matter content and composition of the soil, thereby enhancing its ability to remove Cr(VI) and Cd(II) (Khater et al., 2023). In addition, studies have shown that microorganisms such as *Acidobacteria* and *Myxomycota* can improve the soil environment by regulating soil pH, decomposing complex organic matter, etc., providing more effective conditions for the removal of Cr(VI) and Cd(II) in nZVI@HA (Qi et al., 2024). Some researchers have found that firmicutes can directly participate in the biotransformation of Cr(VI) and Cd(II), and assist nZVI@HA in fixing Cr(VI) and Cd(II) in the soil through biological precipitation, adsorption, or other mechanisms (Chaudhari et al., 2022). At the same time, the metabolic activities of microorganisms (such as *Sphingomonadales* and *Xanthomonadales*) may promote electron transfer processes on the nZVI@HA surface (Yao et al., 2023), accelerating the reduction of Cr(VI) and the removal of Cd(II), thereby enhancing the efficiency of soil remediation through synergistic effects between microorganisms and nZVI@HA.

#### 3.5.2 Rarefaction curves

By high-throughput sequencing of 12 samples in four groups, CK, S7, S8, and S9, the Rarefaction curves of bacterial communities in the soil samples were obtained as shown in **Supplementary Figure S2**, where the horizontal axis represents the sequencing depth and the vertical axis represents the median value of the alpha diversity index calculated 10 times with the box plot. The smoothness of the curve reflects the extent to which sequencing depth affects the diversity of the observed samples.

The saturating trend observed in all four curves demonstrates that the sequencing effort was robust, providing a comprehensive representation of the microbial diversity within the samples (Bal et al., 2016). The size relationship of OTUs in the graph is S9 > S8 > S7 > CK, which indicates that S9 has the highest species richness and further confirms that the addition of nZVI@HA enriches the diversity of soil microorganisms. The species richness of S9 is greater than that of S7 and S8, which may be due to the mutual promotion between Cr (VI) and Cd(II) degradation in the process of nZVI@HA remediation of complex contaminated soil.

#### 3.5.3 Alpha diversity analysis

In order to conduct a comprehensive assessment of the Alpha diversity of the microbial community, the microbial richness is characterized by the Chao1 and Observed species indices (Yanjun et al., 2022), the microbial diversity is characterized by the Shannon index (Luo et al., 2019), the evolutionary-based diversity is characterized by the Faith_pd index (Liu et al., 2023), and the evenness is characterized by the Pielou’s evenness index (Novais et al., 2018).

According to the data in **Table 3**, following nZVI@HA addition, the significant increase in both Chao1 and Observed species indices in groups S7, S8, and S9 (compared to CK, p < 0.05) reflected a corresponding increase in soil microbial richness. It is notable that when comparing within the S7, S8 and S9 groups, it can be concluded that the size relationship of Chao1 and Observed species indices in the samples is: S9 > S8 > S7. This result corresponds to the size relationship of OTUs in the rarefaction curve, which further demonstrates the mutual promotion between Cr (VI) and Cd(II) degradation. Similarly, the Faith_pd index in the S7, S8 and S9 groups was significantly higher than that in the CK group (p < 0.05), suggesting that the addition of nZVI@HA enriched the evolutionary diversity of the microbial community in the soil. The Pielou_e index in the CK group was slightly higher than that in the S7, S8 and S9 groups, suggesting uneven microbial community growth in the soil, which might be caused by microbial community growth in nZVI@HA pores. The Shannon index in CK was slightly lower than that of the S7, S8 and S9 groups, further demonstrating that the addition of nZVI@HA reduced soil exposure to Cr (VI) and Cd(II) environmental stress and promoted soil microbial diversity.

**Table 3:** Alpha diversity index of soil microbial community.

Sample	Chao1	Faith_pd	Observed_species	Pielou_e	Shannon
CK	1502.64±385.74^d^	103.88±17.49^d^	1327.55±245.85^d^	0.96±0.05^a^	7.17±0.35^b^
S7	3266.15±137.04^c^	197.07±35.38^c^	2969.8±671.48^c^	0.84±0.01^b^	9.66±0.34^a^
S8	3772.47±262.19^b^	209.1±11.79^b^	3139.7±280.1^b^	0.81±0.01^b^	9.42±0.09^a^
S9	4286.84±55.36^a^	231.49±15.04^a^	3511.87±218.72^a^	0.82±0.01^b^	9.69±0.17^a^

#### 3.5.4 Beta diversity analysis

**Figure 5:**
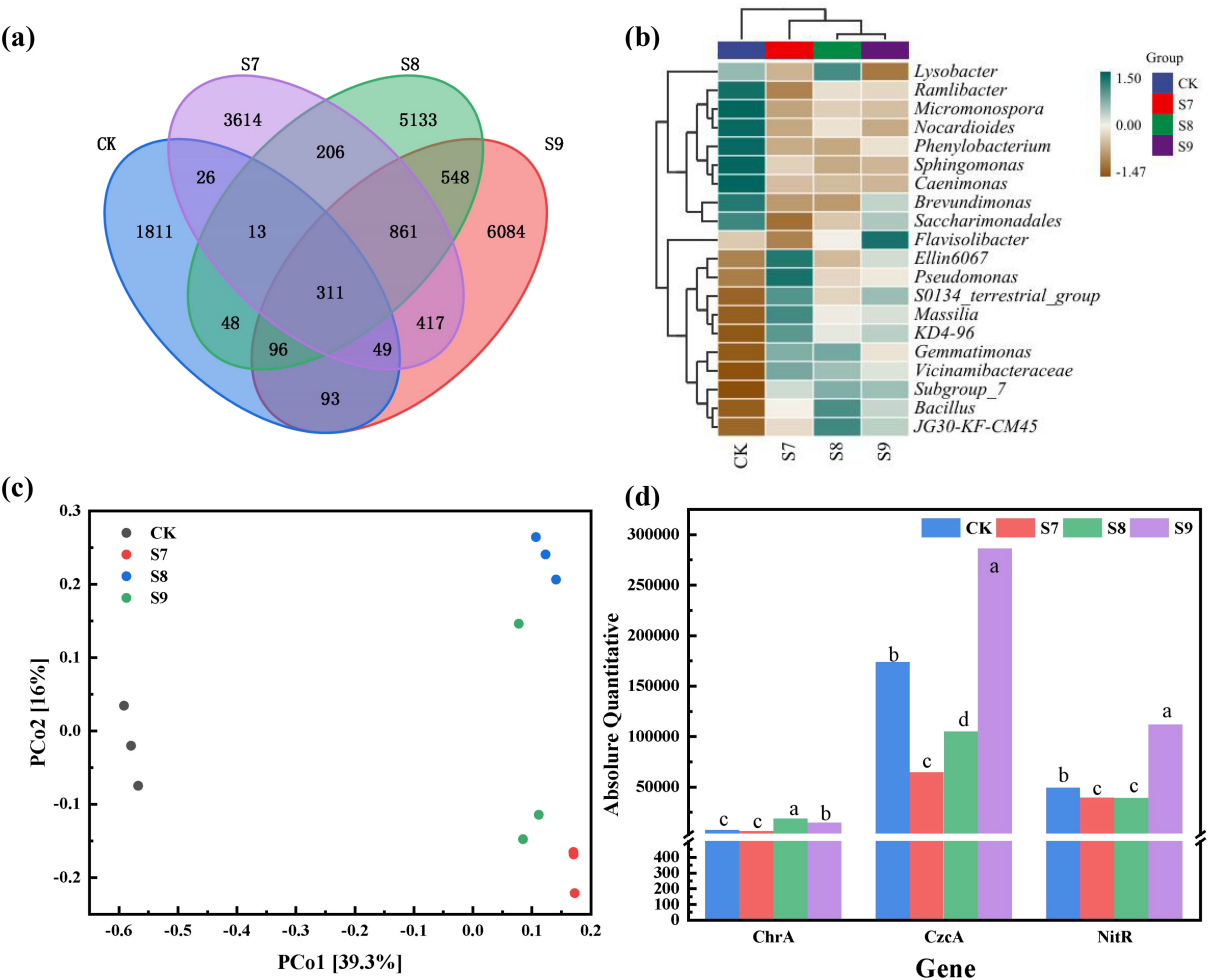
ASV/OUTs Venn diagram of microbial communities **(a)**, cluster heat map analysis of species composition at genus level of soil samples **(b)**, Analysis of bacterial community PcoA in soil samples from different treatments **(c)**, absolute expression levels of *chrA*, *czcA* and *nitR* functional genes in different soil samples(d). (Different lowercase letters represent significant differences between different treatments of the same gene, p<0.05).

To further understand the similarities or differences in community composition in soil samples under treatments CK, S7, S8, and S9, PCoA analysis was performed on each group of samples, as shown in **Figure 5c**, where each point represents a soil sample and points of different colors represent different treatment groups. It can be seen from the figure that the proportion of soil sample variance data that can be explained by the main axis PCo1 is 39.3%, and that explained by the second axis PCo2 is 16%. Compared with the CK group, the projections of the S7, S8 and S9 treatments on the main axis PCo1 are closer, indicating that the microbial community composition of S7, S8 and S9 is similar.

#### 3.5.5 Analysis of ASV/OTU Venn diagrams and species composition clustering heat maps

To study which species are common and which are unique among the four groups of samples CK, S7, S8 and S9, and to present the distribution trend of species abundance in each sample, ASV/OUT Venn diagrams were used for community analysis, as shown in **Figure 5a**. According to the ASV/OUT Venn diagram, the total number of OTUs for the four groups of samples CK, S7, S8 and S9 is 19,310, with 2,447, 5,497, 7,216 and 8,459 respectively. Among them, 311 OTUs were detected in common. Compared with CK, the total number of OTUs in S7, S8 and S9 samples increased significantly, indicating that nZVI@HA is conducive to the development of soil microbial diversity. The total number of OTUs in S7, S8 and S9 samples increased sequentially, indicating that nZVI@HA removed more Cr-Cd co-contamination than single pollution in the soil, which may have reduced the environmental stress of Cr(VI) and Cd(II) in the soil under the synergy of microorganisms and nZVI@HA.

For further comparison of species composition differences at the genus level among CK, S7, S8 and S9 samples, a species composition analysis was performed using a cluster heat map. As shown in **Figure 5b**, there are significant differences in microbial structure in the CK, S7, S8, and S9 treatments, which are highly similar to the results of microbial community composition analysis, relative abundance analysis, and PCR analysis.

### 3.6 nZVI@HA effects on the expression of functional genes in soil microbial communities

The primer information used in the fluorescence quantitative PCR analysis is shown in **Supplementary Table 2**. Quantitative PCR analysis revealed that nZVI@HA significantly affected the expression of *ChrA* (Dai et al., 2019)(chromium resistance-related gene), *CzcA* (Chai et al., 2018)(cadmium resistance-related gene), and *NitR* (Gu et al., 2020) (chromium reduction-related gene) functional genes in soil samples (**Figure 5d**). The gene abundances of Cr resistance-related gene C*hrA* were 6819.85, 5769.17, 18,212.33 and 13,922.99 (gene copies g^-1^ soil) between different treatments CK, S7, S8 and S9, respectively. There were significant differences (p < 0.05) in the Cr resistance gene *ChrA* between S8 and S9 and between S7 and S9, suggesting that the addition of nZVI@HA promoted the growth and reproduction of certain chromium-resistant microorganisms. The gene abundance of the Cd resistance-related gene *CzcA* was 173,417.26, 64,230.10, 104,457.56 and 286,135.59 respectively. Compared with CK, the gene abundance of Cd-resistance gene *CzcA* in S7 and S8 was significantly reduced, while that in S9 was significantly increased. This may suggest that the expression of the *CzcA* gene is suppressed by Cd(II) stress (Dai et al., 2019). The gene abundances of the Cr reduction-related gene *NitR* were 48,859.20, 38,752.51, 38,542.15, and 111,386.63, with a significant difference between CK and S9 (p <0.05). Cr reduction-associated enzyme *NitR* may encode a nicotinamide adenine dinucleotide phosphate (NADPH)-dependent class I chromic acid reductase flavin mononucleotide (FMN) reductase (Chai et al., 2018) that can enhance Cr(VI) and Cd(II) immobilization in soil (Wei et al., 2019; Zhao et al., 2023).

The Alpha diversity results indicated that the addition of nZVI@HA increased species richness and enriched the evolutionary diversity of microbial communities in the soil, which was consistent with the gene expression results of *ChrA* (chromium resistance gene), *CzcA* (cadmium resistance gene), and *NitR* (chromium reduction gene). The expression of these genes not only reflects the microbial response to heavy metal stress, but may be associated with the remediation efficiency of nZVI@HA. The high expression of the *ChrA* gene indicates that the soil microbial community has strong chromium resistance, which helps maintain soil ecological balance. Meanwhile, nZVI@HA may further promote the growth of *ChrA* gene-expressing microorganisms by reducing Cr(VI) to less toxic Cr(III), thereby enhancing the immobilization efficiency of Cr(VI). *CzcA* genes are involved in the cadmium excretion mechanism, and the increase in their expression may be the result of microbial adaptation to cadmium stress. The addition of nZVI@HA reduces the toxicity of Cd(II) to microorganisms, thereby reducing the expression requirement of *CzcA* genes, which is beneficial for maintaining the diversity and functionality of microbial communities. Enzymes encoded by the *NitR* gene are involved in the reduction of Cr(VI), which is crucial for the chemical cycle of Cr(VI). The presence of nZVI@HA may provide additional reduction capacity to assist *nitR* gene-expressing microorganisms in reducing Cr(VI), thereby enhancing the immobilization efficiency of Cr(VI) during soil remediation.

### 3.7 Mechanisms of immobilization of Cr(VI) and Cd(II) in soil by nZVI@HA

In summary, we propose that the mechanisms underlying the removal of Cr(VI) and Cd(II) from soil by humic acid-supported nanoscale zero-valent iron (nZVI@HA) primarily involve the following aspects: chemical reduction, adsorption, ion exchange, and microbe-mediated processes.

Chemical reduction: nZVI@HA is capable of reducing highly toxic Cr(VI) to less toxic Cr(III). This process is primarily driven by the strong reducing capacity of nZVI, which consequently decreases the bioavailability and toxicity of Cr(VI). Concurrently, the presence of humic acid (HA) contributes to maintaining the reactivity and stability of the nZVI. Indirect effects on Cd(II): Although the direct reduction of Cd(II) by nZVI is negligible, the oxidation products generated during the reduction process, such as Fe(II) and Fe(III), may form insoluble complexes or precipitates with Cd(II), thereby indirectly promoting the immobilization of Cd(II) in the soil.

Adsorption: As a natural organic matter, HA possesses abundant functional groups (e.g., carboxyl and phenolic hydroxyl groups) capable of adsorbing Cr(VI) and Cd(II) in the soil. Through adsorption, these functional groups can form stable complexes with Cr(III) and Cd(II), facilitating the removal of these heavy metal ions.

Ion exchange: HA can interact with heavy metal ions in the soil via ion exchange mechanisms, further promoting the removal or stabilization of heavy metals such as Cd(II).

Microbe-mediated processes: The expression of functional genes such as *ChrA* (associated with Cr resistance), *CzcA* (associated with Cd resistance), and *NitR* (associated with Cr reduction) reflects the response of soil microorganisms to heavy metal stress and their capacity to participate in heavy metal biotransformation. The application of nZVI@HA may be associated with an increased relative abundance of microorganisms carrying these functional genes, suggesting a potential microbial adaptation to Cr(VI) and Cd(II) stress. In addition, nZVI@HA can modify soil physicochemical properties and influence the concentration and speciation of heavy metals, which may further reshape the soil microbial community structure. These microbial changes may be indirectly related to the reduced bioavailability and enhanced immobilization of Cr and Cd; however, their causal role in heavy metal transformation requires further experimental verification.

The different performance of nZVI@HA under single-metal and Cr,Cd-contaminated conditions may be related to the distinct chemical behaviors of Cr(VI) and Cd(II) in soil. Cr(VI) mainly exists as mobile oxyanions, whereas Cd(II) is generally present as a divalent cation. Therefore, when Cr and Cd coexist, they may interact differently with HA functional groups, Fe corrosion products, and soil mineral surfaces. The reduction of Cr(VI) to Cr(III) by nZVI may generate less mobile Cr species and newly formed Fe-associated reactive surfaces, which could further provide adsorption or precipitation sites for Cd(II). Meanwhile, Cd(II) may compete for binding sites on HA, Fe oxides, and soil colloids, thereby altering the immobilization behavior of Cr compared with single-metal contamination.

In conclusion, the removal of Cr and Cd from soil by nZVI@HA is driven by the aforementioned mechanisms, which encompass both direct physicochemical interactions and the involvement of biological processes. This synergistic mechanism renders nZVI@HA a highly efficient and environmentally friendly material for soil remediation, offering an effective strategy for the restoration of co-contaminated soils.

## 4 Conclusion

The remediation effects of HA, nZVI and nZVI@HA on Cr-, Cd- and Cr,Cd-contaminated soil were investigated. nZVI@HA effectively reduces the mobility and bioavailability of Cr and Cd in the soil by promoting their transformation from exchangeable forms to residual, Fe-Mn oxide-bound, and carbonate-bound.The nZVI@HA altered the relative abundance of microbial taxa and functional genes potentially associated with Cr(VI) and Cd(II) resistance and transformation, such as *ChrA*, *CzcA*, and *NitR*. Meanwhile, nZVI@HA improved soil physicochemical properties and promoted the transformation of Cr and Cd into less bioavailable fractions, which may be associated with shifts in microbial community structure and may indirectly contribute to enhanced metal immobilization.Given that this study was limited to a small-scale laboratory experiment on soil cultivation, the results may differ from actual field conditions. Future studies should further conduct pot and field experiments with representative crops to evaluate the long-term stability of Cr and Cd immobilization, the potential remobilization risk under rhizosphere conditions, and the agronomic and ecological safety of nZVI@HA application.

## Data Availability

The original contributions presented in the study are included in the article/Supplementary Material. Further inquiries can be directed to the corresponding author.

## References

[B1] Algethami J. S. Ibrahim M. Javed W. Alosaimi E. H. Irshad M. K. (2024). Efficacy of Fe-BC in enhancing growth, photosynthesis, nutrition, and alleviating the toxicity of Cd and Cr in rapeseed (Brassica napus L.): A tool for managing the environment and attaining sustainable agriculture. Environ. Technol. Innovation 36, 103789. doi: 10.1016/j.eti.2024.103789 38826717

[B2] Bal J. Yun S.-H. Yeo S.-H. Kim J.-M. Kim B.-T. Kim D.-H. (2016). Effects of initial moisture content of Korean traditional wheat-based fermentation starter nuruk on microbial abundance and diversity. Appl. Microbiol. Biotechnol. 101 (5), 2093–2106. doi: 10.1007/s00253-016-8042-2 27975136

[B3] Boysan Canal S. (2025). A synergistic biochar-mycorrhizal strategy enhances rapeseed phytoremediation and antioxidant defences under Cd and Cr stress. Sci. Rep. (Nature Publisher Group) 15, 37917. doi: 10.1038/s41598-025-22959-3 41162672 PMC12572378

[B4] Canal S. B. Bozkurt M. A. Yilmaz H. (2022). Effects of Humic Acid and EDTA on Phytoremediation, Growth and Antioxidant Activity in Rapeseed (Brassica napus L.) Grown under Heavy Metal Stress. Pol. J. Environ. Stud. 31, 4051–4060. doi: 10.15244/pjoes/148120 41579550

[B5] Cezar R. M. Vezzani F. M. Joris H. A. W. Barth G. Kaschuk G. (2025). Crop rotation restructures soil microbial communities in Subtropical Oxisol, South Brazil. Sci. Agric. 82, e20240134. doi: 10.1590/1678-992x-2024-0134 41099703

[B6] Chai L. Ding C. Tang C. Yang W. Yang Z. Wang Y. . (2018). Discerning three novel chromate reduce and transport genes of highly efficient Pannonibacter phragmitetus BB: From genome to gene and protein. Ecotoxicol. Environ. Saf. 162, 139–146. doi: 10.1016/j.ecoenv.2018.06.090 29990725

[B7] Chaudhari A. Paul D. Thamke V. Bagade A. Bapat V. A. Kodam K. M. (2022). Concurrent removal of reactive blue HERD dye and Cr(VI) by aerobic bacterial granules. J. Cleaner Prod. 367, 133075. doi: 10.1016/j.jclepro.2022.133075 38826717

[B8] Chen A. Huang Y. Liu H. (2023). Fabrication of Chitin microspheres supported sulfidated nano zerovalent iron and their performance in Cr (VI) removal. Chemosphere. 323, 139609. doi: 10.1016/j.chemosphere.2023.139609 37482322

[B9] Chen X. Ji D. Wang X. Zang L. (2017). “ Review on nano zerovalent iron (nZVI): from modification to environmental applications,” in IOP Conference Series: Earth and Environmental Science. (Bristol: IOP Publishing Ltd.).

[B10] Chen X. Li X. Xu D. Yang W. Bai S. (2020). Application of nanoscale zero-valent iron in hexavalent chromium-contaminated soil: A review. Nanotechnol. Rev. 9, 1222–1233. doi: 10.1515/ntrev-2020-0059 31755547

[B11] Dai M. Zhou G. Ng H. Y. Zhang J. Wang Y. Li N. . (2019). Diversity evolution of functional bacteria and resistance genes (CzcA) in aerobic activated sludge under Cd(II) stress. J. Environ. Manage. 251, 109519. doi: 10.1016/j.jenvman.2019.109519 31514000

[B12] Dai Y. Jing Z. Li Z. Zhu Y. Qiu F. Pan J. . (2022). Efficient cellulose aerogel biosorbents functionalized by nanosized zero-valent iron: Isotherms, kinetics, thermodynamics and mechanism of tellurium adsorption. Colloids Surfaces A. Physicochem. Eng. Aspects. 651, 129765. doi: 10.1016/j.colsurfa.2022.129765 38826717

[B13] Daulton T. L. Little B. J. Lowe K. Jones-Meehan J. (2020). Microbial reduction of chromium(VI): in-situ environmental cell transmission electron microscopy study using electron energy loss spectroscopy. Microsc. Microanal. 26(S2), 1994–1995. doi: 10.1017/s143192760002674x 12597792

[B14] Dong H. Deng J. Xie Y. Zhang C. Jiang Z. Cheng Y. . (2017). Stabilization of nanoscale zero-valent iron (nZVI) with modified biochar for Cr(VI) removal from aqueous solution. J. Hazard. Mater. 332, 79–86. doi: 10.1016/j.jhazmat.2017.03.002 28285109

[B15] Francioso O. López-Tobar E. Torreggiani A. Iriarte M. Sanchez-Cortes S. (2019). Stimulated adsorption of humic acids on capped plasmonic ag nanoparticles investigated by surface-enhanced optical techniques. Langmuir. 35, 11040–11048. doi: 10.1021/acs.langmuir.9b00190 30762359

[B16] Fu R. Zhang X. Xu Z. Guo X. Bi D. Zhang W. (2016). Fast and highly efficient removal of chromium (VI) using humus-supported nanoscale zero-valent iron: Influencing factors, kinetics and mechanism. Sep. Purif. Technol. 171, 62–69. doi: 10.1016/j.seppur.2016.10.058 38826717

[B17] Gholami L. Rahimi G. (2023). The efficiency of potato peel biochar for the adsorption and immobilization of heavy metals in contaminated soil. Int. J. Phytorem. 25, 263–273. doi: 10.1080/15226514.2022.2073962 35579507

[B18] Gil-Díaz M. Pinilla P. Alonso J. Lobo M. C. (2017). Viability of a nanoremediation process in single or multi-metal(loid) contaminated soils. J. Hazard. Mater. 321, 812–819. doi: 10.1016/j.jhazmat.2016.09.071 27720472

[B19] Gómez-Sagasti M. T. Epelde L. Anza M. Urra J. Alkorta I. Garbisu C. (2018). The impact of nanoscale zero-valent iron particles on soil microbial communities is soil dependent. J. Hazard. Mater. 364, 111–119. doi: 10.1016/j.jhazmat.2018.10.034 30390579

[B20] Gopinath K. P. Rajagopal M. Krishnan A. Sreerama S. K. (2021). A review on recent trends in nanomaterials and nanocomposites for environmental applications. Curr. Anal. Chem. 17, 1328–1345. doi: 10.2174/1573411016666200102112728

[B21] Gu R. Gao J. Dong L. Liu Y. Li X. Bai Q. . (2020). Chromium metabolism characteristics of coexpression of ChrA and ChrT gene. Ecotoxicol. Environ. Saf. 204, 111060. doi: 10.1016/j.ecoenv.2020.111060 32768747

[B22] Guo Y. Li X. Liang L. Lin Z. Su X. Zhang W. (2021). Immobilization of cadmium in contaminated soils using sulfidated nanoscale zero-valent iron: Effectiveness and remediation mechanism. J. Hazard. Mater. 416, 126605. doi: 10.1016/j.jhazmat.2021.126605 34329110

[B23] Haider F. U. Ain N. Khan I. Farooq M. Habiba Cai L. . (2024). Co-application of biochar and plant growth regulators improves maize growth and decreases Cd accumulation in cadmium-contaminated soil. J. Cleaner Prod. 440, 140515. doi: 10.1016/j.jclepro.2023.140515 38826717

[B24] Hou S. Wu B. Peng D. Wang Z. Wang Y. Xu H. (2019). Remediation performance and mechanism of hexavalent chromium in alkaline soil using multi-layer loaded nano-zero-valent iron. Environ. pollut. 251, 702–710. doi: 10.1016/j.envpol.2019.05.083 31181500

[B25] Jaiswal A. Verma A. Jaiswal P. (2018). Detrimental effects of heavy metals in soil, plants, and aquatic ecosystems and in humans. J. Environ. Pathol. Toxicol. Oncol. 37(4), 353–366. doi: 10.1615/jenvironpatholtoxicoloncol.2018025348 30317970

[B26] Jia H. Muhae-Ud-Din G. Zhang H. Zong Q. Zhao S. Guo Q. . (2022). Characterization of rhizosphere microbial communities for disease incidence and optimized concentration of difenoconazole fungicide for controlling of wheat dwarf bunt. Front. Microbiol. 13, 853176. doi: 10.3389/fmicb.2022.853176 35615520 PMC9125210

[B27] Jiang Y. Yang F. Dai M. Ali I. Shen X. Hou X. . (2021). Application of microbial immobilization technology for remediation of Cr(VI) contamination: A review. Chemosphere. 276, 130170. doi: 10.1016/j.chemosphere.2021.131721 34352550

[B28] Jin Y. Wang Y. Li X. Luo T. Ma Y. Wang B. . (2023a). Remediation and its biological responses to Cd(II)-Cr(VI)-Pb(II) multi-contaminated soil by supported nano zero-valent iron composites. Sci. Total Environ. 867, 161344. doi: 10.1016/j.scitotenv.2022.161344 36610630

[B29] Jin Y. Wang Y. X. Li X. Luo T. Ma Y. S. Wang B. . (2023b). Remediation and its biological responses to Cd(II)-Cr(VI)-Pb(II) multi-contaminated soil by supported nano zero-valent iron composites. Sci. Total Environ. 867, 161344. doi: 10.1016/j.scitotenv.2022.161344 36610630

[B30] Jingzi B. Daniel C. W. T. Nanthi S. B. Kitae B. Yong Sik O. Xiang-Dong L. (2018). Interactions of food waste compost with metals and metal-chelant complexes during soil remediation. J. Cleaner Prod. 197, 1021–1030. doi: 10.1016/j.jclepro.2018.04.239 38826717

[B31] Kalubi K. N. Mehes-Smith M. Spiers G. Omri A. (2017). Variation in whole DNA methylation in red maple (Acer rubrum) populations from a mining region: association with metal contamination and cation exchange capacity (CEC) in podzolic soils. Ecotoxicology. 26, 1213–1224. doi: 10.1007/s10646-017-1773-8 28204976

[B32] Khalil A. M. E. Eljamal O. Amen T. W. M. Sugihara Y. Matsunaga N. (2018). Scrutiny of interference effect of ions and organic matters on water treatment using supported nanoscale zero-valent iron. Environ. Earth Sci. 77, 563. doi: 10.1007/s12665-018-7661-6 30311153

[B33] Khater D. Z. Amin R. S. Fetohi A. E. Mahmoud M. El-Khatib K. M. (2023). Insights on hexavalent chromium(VI) remediation strategies in abiotic and biotic dual chamber microbial fuel cells: electrochemical, physical, and metagenomics characterizations. Sci. Rep. 13, 20364, doi: 10.1038/s41598-023-47450-9 37978236 PMC10656525

[B34] Kumar V. Dwivedi S. K. Oh S. (2022). A review on microbial-integrated techniques as promising cleaner option for removal of chromium, cadmium and lead from industrial wastewater. J. Water Process Eng. 49, 103044. doi: 10.1016/j.jwpe.2022.102727 38826717

[B35] Liang W. Shen Y. Chen X. Cai D. Wang D. Luo K. . (2022). Enhanced simultaneous removal of Cr(VI) and Cd(II) from aqueous solution and soil: a novel carbon microsphere–calcium alginate supported sulfide-modified nZVI composite. Environ. Sci. Nano. 9, 3471–3484. doi: 10.1039/D2EN00483F

[B36] Liron F. Kartik C. Dror A. Edris T. Amanda K.-H. Hadas M. (2022). Accelerating microbial activity of soil aquifer treatment by hydrogen peroxide. Energies. 15(11), 3852. doi: 10.3390/en15113852 30654563

[B37] Liu Y. Bao L. Sun W. Cui Y. Li X. Ji X. . (2023). Diversity and composition of active and total bacteria in rhizospheric soil in response to continuous cropping years of Panax notoginseng. Folia Microbiol. 68, 527–538. doi: 10.1007/s12223-023-01109-0 38038798

[B38] Liu Z. Nan Z. Zhao C. Yang Y. (2018). Potato absorption and phytoavailability of Cd, Ni, Cu, Zn and Pb in sierozem soils amended with municipal sludge compost. J. Arid. Land. 10, 778–789. doi: 10.1007/s40333-018-0062-6 30311153

[B39] Liu Z. Y. Zhao X. Yang J. W. Chen X. Cai Y. B. Shaaban M. . (2025). Microbial removal mechanism of chromium and cadmium by humic acid-loaded nano zero-valent iron prepared by liquid-phase reduction method. Front. Plant Sci. 16. doi: 10.3389/fpls.2025.1596063 40894499 PMC12392117

[B40] Lopes E. M. G. Mota M. F. C. Santos Júnior J. M. Reis M. M. Frazão L. A. Fernandes L. A. (2024). Biochar alters the soil microbiological activity of sugarcane fields over time. Sci. Agric. 81, e20230289. doi: 10.1590/1678-992x-2023-0289 41099703

[B41] Luo X. Wang M. K. Hu G. Weng B. (2019). Seasonal change in microbial diversity and its relationship with soil chemical properties in an orchard. PloS One. 14(4), e0215556. doi: 10.1371/journal.pone.0215556 31891580 PMC6938340

[B42] Manel C. Omar S. Imen Challougui F. Souhir A. Ghassen A. Moez J. . (2016). Characterization of efficient plant-growth-promoting bacteria isolated from Sulla coronaria resistant to cadmium and to other heavy metals. C.R. Biol. 339, 391–398. doi: 10.1016/j.crvi.2016.04.015 27498183

[B43] Novais M. H. Penha A. M. Morales E. A. Potes M. Salgado R. Morais M. (2018). Vertical distribution of benthic diatoms in a large reservoir (Alqueva, Southern Portugal) during thermal stratification. Sci. Total Environ. 645, 1252–1262. doi: 10.1016/j.scitotenv.2018.12.251 31096337

[B44] Oladipo O. O. Ayo J. O. Ambali S. F. Mohammed B. (2016). Evaluation of hepatorenal impairments in Wistar rats coexposed to low-dose lead, cadmium and manganese: insights into oxidative stress mechanism. Toxicol. Mech. Methods. 27(1), 1–8. doi: 10.1080/15376516.2016.1223242 27599793

[B45] Peipei S. Fupeng S. Jin L. Wenjing M. Xiaoyu G. (2024). Remediation of cadmium-contaminated soil by biochar-loaded nano-zero-valent iron and its microbial community responses. J. Environ. Chem. Eng. 12, 112311. doi: 10.1016/j.jece.2024.112311 38826717

[B46] Qi L. Jingjing C. Linfeng L. Xiaoyang L. Yichun L. (2024). Soil amendments alter cadmium distribution and bacterial community structure in paddy soils. Sci. Total Environ. 912, 171399. doi: 10.1016/j.scitotenv.2024.171399 38458464

[B47] Ren J. Mi X. Tao L. (2020). Stabilization of cadmium in polluted soil using palygorskite-coated nanoscale zero-valent iron. J. Soils Sediments. 20, 3918–3928. doi: 10.1007/s11368-020-02841-7 30311153

[B48] Sarkis L. F. Rabêlo F. H. S. Hippler F. W. R. Guelfi D. (2024). Soil organic matter influences the agronomic efficiency of boron fertilizers in sandy Oxisol cultivated with soybean. Sci. Agric. 81, e20230231. doi: 10.1590/1678-992x-2023-0231 41099703

[B49] Shi Y. Yu Y. Xiang M. Cui P. Cui J. Zhang F. . (2023). Changes in molybdenum bioaccessibility in four spiked soils with respect to soil pH and organic matter. J. Environ. Manage. 337, 117476. doi: 10.1016/j.jenvman.2023.117476 36773452

[B50] Silva R. C. Guimarães C. C. B. Azevedo A. C. Alves M. R. Demattê J. A. M. (2024). Precipitation of amorphous iron and aluminum during the weathering of rock dust in soil columns. Sci. Agric. 81, e20230219. doi: 10.1590/1678-992x-2023-0219 41099703

[B51] Sun Y. Zheng F. Wang W. Zhang S. Wang F. (2020). Remediation of Cr(VI)-contaminated soil by nano-zero-valent iron in combination with biochar or humic acid and the consequences for plant performance. Toxics 8, 26. doi: 10.3390/toxics8020026. [Online]. 32260118 PMC7357137

[B52] Tingting L. Yanyan W. Yuefeng Y. Junhui Z. Zengyu Z. Fuhai Z. . (2022). Silkworm excrement reduces cadmium and arsenic accumulation in rice grains by altering soil chemical properties, microbial community, and promoting iron plaque formation. Appl. Soil Ecol. 177, 104710. doi: 10.1016/j.apsoil.2022.104710 38826717

[B53] Wang Y. Liu Y. Su G. Yang K. Lin D. (2021). Transformation and implication of nanoparticulate zero valent iron in soils. J. Hazard. Mater. 418, 125207. doi: 10.1016/j.jhazmat.2021.125207 33513552

[B54] Wang R. Sang P. Guo Y. Jin P. Cheng Y. Yu H. . (2022). Cadmium in food: Source, distribution and removal. Food Chem. 405, 134666. doi: 10.1016/j.foodchem.2022.134666 36335725

[B55] Wang L. Zhou H. Liu J. Chen J. Wei S. Jiang Z. (2018). Effect of humic acid on the nitrate removal by strong base anion exchanger supported nanoscale zero-valent iron composite. Water Air Soil pollut. 229, 349. doi: 10.1007/s11270-018-3988-6 30311153

[B56] Wei X. Yin H. Peng H. Chen R. Lu G. Dang Z. (2019). Reductive debromination of decabromodiphenyl ether by iron sulfide-coated nanoscale zerovalent iron: Mechanistic insights from Fe(II) dissolution and solvent kinetic isotope effects. Environ. pollut. 252, 1536–1544. doi: 10.1016/j.envpol.2019.07.007 31306823

[B57] Xia S. Song Z. Jeyakumar P. Bolan N. Wang H. (2019). Characteristics and applications of biochar for remediating Cr(VI)-contaminated soils and wastewater. Environ. Geochem. Health. 41, 2595–2613. doi: 10.1007/s10653-019-00445-w 31673917

[B58] Xu S. Zhao J. Yu Q. Qiu X. Sasaki K. (2019). Understanding how specific functional groups in humic acid affect the sorption mechanisms of different calcinated layered double hydroxides. Chem. Eng. J. 375, 123633. doi: 10.1016/j.cej.2019.123633 38826717

[B59] Yan T. Xue J. Zhou Z. Wu Y. (2021). Biochar-based fertilizer amendments improve the soil microbial community structure in a karst mountainous area. Sci. Total Environ. 776, 148757. doi: 10.1016/j.scitotenv.2021.148757 34225142

[B60] Yang J. Wang S. Xu N. Ye Z. Yang H. Huangfu X. (2021). Synthesis of montmorillonite-supported nano-zero-valent iron via green tea extract: Enhanced transport and application for hexavalent chromium removal from water and soil. J. Hazard. Mater. 403, 123560. doi: 10.1016/j.jhazmat.2021.126461 34186421

[B61] Yang Y. Ye C. Zhang W. Zhu X. Li H. Yang D. . (2023). Elucidating the impact of biochar with different carbon/nitrogen ratios on soil biochemical properties and rhizosphere bacterial communities of flue-cured tobacco plants. Front. Plant Sci. 14, 1250669. doi: 10.3389/fpls.2023.1250669 37790782 PMC10543665

[B62] Yanjun L. Jing Z. Zhisong F. Liang L. Yudong W. Li F. . (2022). The microbial characteristics of patients with esophageal squamous cell carcinoma after chemotherapy and after immunotherapy. J. Clin. Oncol. 40(16_suppl), e16049. doi: 10.1200/jco.2022.40.16_suppl.e16049 42148471

[B63] Yao B. Liu M. Tang T. Hu X. Yang C. Chen Y. (2023). Enhancement of anaerobic digestion of ciprofloxacin wastewater by nano zero-valent iron immobilized onto biochar. Bioresour. Technol. 376, 129462. doi: 10.1016/j.biortech.2023.129462 37429552

[B64] Yao B. Liu Y. Zou D. (2019). Removal of chloramphenicol in aqueous solutions by modified humic acid loaded with nanoscale zero-valent iron particles. Chemosphere. 230, 616–623. doi: 10.1016/j.chemosphere.2019.03.098 30933739

[B65] Yu H. Zhang Z. Zhang Y. Fan P. Xi B. Tan W. (2020). Metal type and aggregate microenvironment govern the response sequence of speciation transformation of different heavy metals to microplastics in soil. Sci. Total Environ. 733, 141956. doi: 10.1016/j.scitotenv.2020.141956 32890822

[B66] Yuan J. C. Liu J. Z. Fan W. Liang Y. Cheng S. Zhang S. M. . (2024). Yield benefit and soil fertility improved by different fertilizer application placements and supplementary organic manure in maize (Zea mays L.). Sci. Agric. 81, e20230057. doi: 10.1590/1678-992x-2023-0057 41099703

[B67] Zhang Q. Qin Z. Xiahou J. Li Y. Yan Y. Feng X. . (2023). Effects and mechanisms of Al substitution on the catalytic ability of ferrihydrite for Mn(II) oxidation and the subsequent oxidation and immobilization of coexisting Cr(III). J. Hazard. Mater. 452, 131351. doi: 10.1016/j.jhazmat.2023.131351 37027918

[B68] Zhao K. Zhang W. Liang Z. Zhao H. Chai J. Yang Y. . (2023). Facilitating new chromium reducing microbes to enhance hexavalent chromium reduction by in situ sonoporation-mediated gene transfer in soils. Environ. Sci. Technol. 57, 17353–17363. doi: 10.1021/acs.est.3c04655 37747805

[B69] Zhu X. F. Liu J. D. Li L. Zhen G. Y. Lu X. Q. Zhang J. . (2023). Prospects for humic acids treatment and recovery in wastewater: A review. Chemosphere 312, 137193. doi: 10.1016/j.chemosphere.2022.137193 36370766

