# Supplementary material for: A humic acid loaded nano-zero-valent iron composite for enhanced immobilization of chromium and cadmium from soil
Source: Front Plant Sci. 2026 Jul 27;17:1894863. doi: 10.3389/fpls.2026.1894863 (PMC13454282; doi:10.3389/fpls.2026.1894863)
Supplement: Supplementary file 1 [file DataSheet1.pdf]

Supplementary Material

1 Supplementary Data

Text S1:

Preparation of humic acid-loaded NZVI

The NZVI@HA (HA:Fe(Ⅱ)=3:1)was synthesized through liquid-phase reduction. Purified HA and ferrous sulfate heptahydrate (0.05mol/L $FeSO_4 \cdot 7H_2O$) were pooled in 75 mL of anaerobic water (adjusted pH of the solution was 4.0) and agitated on an electric shaker at 150 rpm for 24 h at 25℃.After that, the solution was moved into a three-neck round bottom flask which already had a volume of 25 mL of ethanol. The N_2 gas was purged into the solution, while concurrently, a robust stirring was performed for 30 min to remove dissolved O_2. Then, $NaBH_4$(0.2mol/L) solution was mixed dropwise into the slurry under strong stirring conditions. The reaction continued under a nitrogen atmosphere for 10 minutes to ensure complete reduction of Fe(II) to Fe^0, resulting in a solution with a black precipitate.The three-necked flask was sealed under nitrogen protection, and the solution was separated by magnet adsorption. The supernatant was decanted, and the resulting precipitates were washed thrice with deoxygenated pure water and thrice with anhydrous ethanol. Finally, it was dried under vacuum at 60℃ for 12 hours, and then stored for future use.

Text S2:

Supplementary soil information

The tested soil was mainly classified as heavy loam to light loam, with relatively high clay content, strong water and nutrient retention capacity, and comparatively low permeability. the particle composition is dominated by silt and clay, with sand (>0.075 mm), silt (0.005 – 0.075 mm), and clay (<0.005 mm) accounting for approximately 2.1%, 47.4%, and 50.5%, According to the following WRB Soil Taxonomy, based on the sampling area of the soil and its existing physical and chemical properties, this soil type is classified as Anthrosols.

WRB Soil Taxonomy

WRB Soil Taxonomy	General interpretation
Histosols	Organic soils
Anthrosols	Human-modified agricultural soils
Technosols	Soils strongly influenced by technical/artificial materials
Cryosols	Soils affected by permafrost
Leptosols	Very shallow or stony soils
Solonetz	Sodium-rich alkaline soils
Vertisols	Shrink–swell clay soils
Solonchaks	Salt-affected soils
Gleysols	Waterlogged soils with reducing conditions
Andosols	Volcanic ash soils

WRB Soil Taxonomy	General interpretation
Podzols	Strongly leached acidic soils with spodic horizons
Plinthosols	Soils with iron-rich plinthite or hard iron concretions
Nitisols	Deep, well-structured, clay-rich tropical soils
Ferralsols	Highly weathered tropical soils rich in iron and aluminum oxides
Planosols	Soils with a dense subsurface horizon causing seasonal water stagnation
Stagnosols	Soils affected by prolonged water stagnation
Chernozems	Dark, humus-rich steppe soils
Kastanozems	Chestnut-colored dry steppe soils
Phaeozems	Dark, humus-rich soils with relatively high base status
Umbrisols	Dark, acidic, humus-rich soils with low base status
Durisols	Soils with silica accumulation or duric horizons
Gypsisols	Soils with gypsum accumulation
Calcisols	Soils with calcium carbonate accumulation
Retisols	Soils with retic or net-like clay depletion features
Acrisols	Strongly leached acidic soils with low-activity clays
Lixisols	Leached soils with low-activity clays and higher base status
Alisols	Acidic soils with high-activity clays and high exchangeable aluminum
Luvisols	Clay-illuvial soils with relatively high base status
Cambisols	Weakly to moderately developed soils
Arenosols	Sandy soils
Fluvisols	Young soils developed from alluvial deposits
Regosols	Weakly developed mineral soils

Text S3:

Methods for simulating contaminated soil on agricultural land

To simulate agricultural soil pollution, in accordance with the "Soil Environmental Quality Risk Control Standard for Soil Contamination of Agricultural Land" (GB15618) issued by the Ministry of Ecology and Environment of the People's Republic of China in 2018, the control values for cadmium (Cd) and chromium (Cr) pollution in agricultural soil are set at 4 mg/kg and 1000 mg/kg, respectively. Based on these limits, artificially contaminated agricultural soil was prepared as follows: sieved soil was mixed with pre-prepared solutions at a soil-to-water ratio of 1:1 (L:kg). The solutions used were 1 g/L $K_2Cr_2O_7$ solution, 4 mg/L $CdCl_2{\cdot}2.5H_2O$ solution, and a mixed solution of $K_2Cr_2O_7$ and $CdCl_2{\cdot}2.5H_2O$. The mixture was thoroughly stirred to ensure uniform slurry consistency, followed by natural aging and air-drying for 3 months. The dried soil was then passed through a 100-mesh nylon sieve and stored for future use.

Text S4：

Soil characteristic analysis

To analyze the changes of soil properties during the soil remediation, the soil pH, soil cation exchange capacity (CEC), soil dissolved organic carbon (DOC), and available cadmium and chromium

were measured at the 1st, 30th, and 50th days of the remediation process.

Determination of soil pH: Refer to the "Determination of Soil pH by Potentiometric Method" (HJ962-2018) issued by the Ministry of Ecology and Environment of the People's Republic of China in 2018. Weigh 2.0 g of soil sample and place it in a 50 mL beaker, add 5 mL of water. Seal the beaker with plastic wrap and vigorously stir with a magnetic stirrer for 2 minutes. After 30 minutes of standing, measure the soil pH using a pH meter within 1 hour.

Determination of Soil Cation Exchange Capacity (CEC): Refer to the "Determination of Soil Cation Exchange Capacity-Extraction with Hexametallic Cobalt Chloride-Spectrophotometry" (HJ889-2017) issued by the Ministry of Ecology and Environment of the People's Republic of China in 2017. Weigh 3.5 g of the homogenized sample and transfer it to a 100 mL centrifuge tube. Add 50.0 mL of hexametallic cobalt chloride solution, tighten the tube seal, and place it on a constant temperature shaker. Oscillate at 20±2°C for 60 minutes, adjusting the shaking frequency to maintain the suspension of the soil extract mixture during the process. Centrifuge at 4000 rpm for 10 minutes, collect the supernatant in a cuvette, and measure its absorbance using a spectrophotometer. Calculate the CEC value according to Formula 1.1.

$$CEC=\frac{(A_0-A)\times V\times 3}{b\times m\times w_{dm}} \quad \text{(Formula 1.1)}$$

In the formula:

CEC : Cation Exchange Capacity of soil samples, $cmol\cdot kg^{-1}$.

A_0 : Absorbance of the blank sample;

A : sample absorbance;

V : volume of extract，mL;

3 : The charge number of $[Co(NH_3)_6]^{3+}$;

b : slope of the standard curve;

m : Sampling quality,g;

w_{dm} : Dry matter content of soil samples,%

Determination of Soil Dissolved Organic Carbon (DOC): Refer to Hussain et al.(Hussain et al., 2020) , who conducted the extraction using ultrapure water under shaking conditions and measured it using a TOC analyzer.

Determination of Available Cadmium and Chromium in Soil: Refer to the "Determination of 8 Available Elements in Soil-Ethylenediaminetetraacetic Acid Extraction-Inductively Coupled Plasma Emission Spectrometry" (HJ804-2016) issued by the Ministry of Ecology and Environment of the People's Republic of China in 2016. Weigh 5.0g (accurate to 0.01g) of the sample and place it in a 100mL Erlenmeyer flask. Add 10.0mL of extraction solution and seal the flask tightly. Under conditions of 20±2℃, oscillate the flask at a frequency of 160 rpm to 200 rpm for 2 hours. Slowly transfer the extraction solution into a centrifuge tube and centrifuge for 10 minutes. The supernatant is then filtered by medium-speed quantitative filter paper under gravity and analyzed within 48 hours. Calculate the available cadmium and chromium in the soil using Formula 1.2.

$$\omega=\frac{(\rho-\rho_0)\times V\times f}{m\times W_{dm}} \quad \text{(Formula 1.2)}$$

In the formula:

ω: The Content of Available Elements in Soil Samples, mg/kg；

ρ: The mass concentration of the available elements in the sample was determined by referring to the standard curve, mg/L；

ρ_0: The mass concentration of the available elements in the blank sample of the laboratory, mg/L；

V: The volume of the extract solution added during sample preparation, mL；

f: The dilution factor of the sample；

m: Weigh the mass of the sample after sieving, g；

w_{dm}: Dry matter content of soil samples, %

Morphological grading: In this study, the Tessier five-step sequential extraction method(Vilar et al., 2005) was adopted to conduct classification extraction of the exchangeable, carbonate-bound, iron-manganese oxide-titanium, combined organic, and residual states of Cr and Cd in the soil.

Text S5

Analysis of soil enzyme activity

The activity of soil peroxidase was determined by the potassium permanganate titration method, with the peroxidase activity (mg/g) expressed as the mass (mg) of H_2O_2 consumed by 1 g of soil sample after 20 minutes of incubation; the activity of soil urease was determined by the phenol sodium-hypochlorite colorimetric method, with the urease activity (mg/g) expressed as the mass (mg) of NH3-N produced by 1 g of soil sample after 24 hours of incubation; the activity of soil sucrose enzyme was determined by the 3,5-dinitrosalicylic acid colorimetric method, with the sucrose enzyme activity (mg/g) expressed as the mass (mg) of glucose generated by 1 g of soil sample after 24 hours of incubation.

2 Supplementary Figures and Tables

Supplementary Tables

Table S1 Soil information

Soil type	pH	Dissolved Organic Carbon ($mg \cdot kg^{-1}$)	Cation Exchange Capacity ($cmol \cdot kg^{-1}$)	Organic matter (g/kg)	Total N (g/kg)
Anthrosols	6.28	200.09	240.87	20.4	1.12

Table S2 Primer information used in this experiment

Gene	Forward primer (5’- 3’)	Reverse primer (5’- 3’)	文献
CzcA	TCGACGGBGCCGTGGTSMTBGTCGAGAA	GTVAWSGCCAKCGGVBGGAACA	(Dai et al., 2019)
NitR	GACACCCGCCCGCATCTCAT	TGTCCCAGTCGCCTTCCACC	(Chai et al., 2018)
ChrA	GAATGCGCCCATGAAACC	TAGCACCTGTCGTTCTGTT	(Gu et al., 2020)

Supplementary Figures

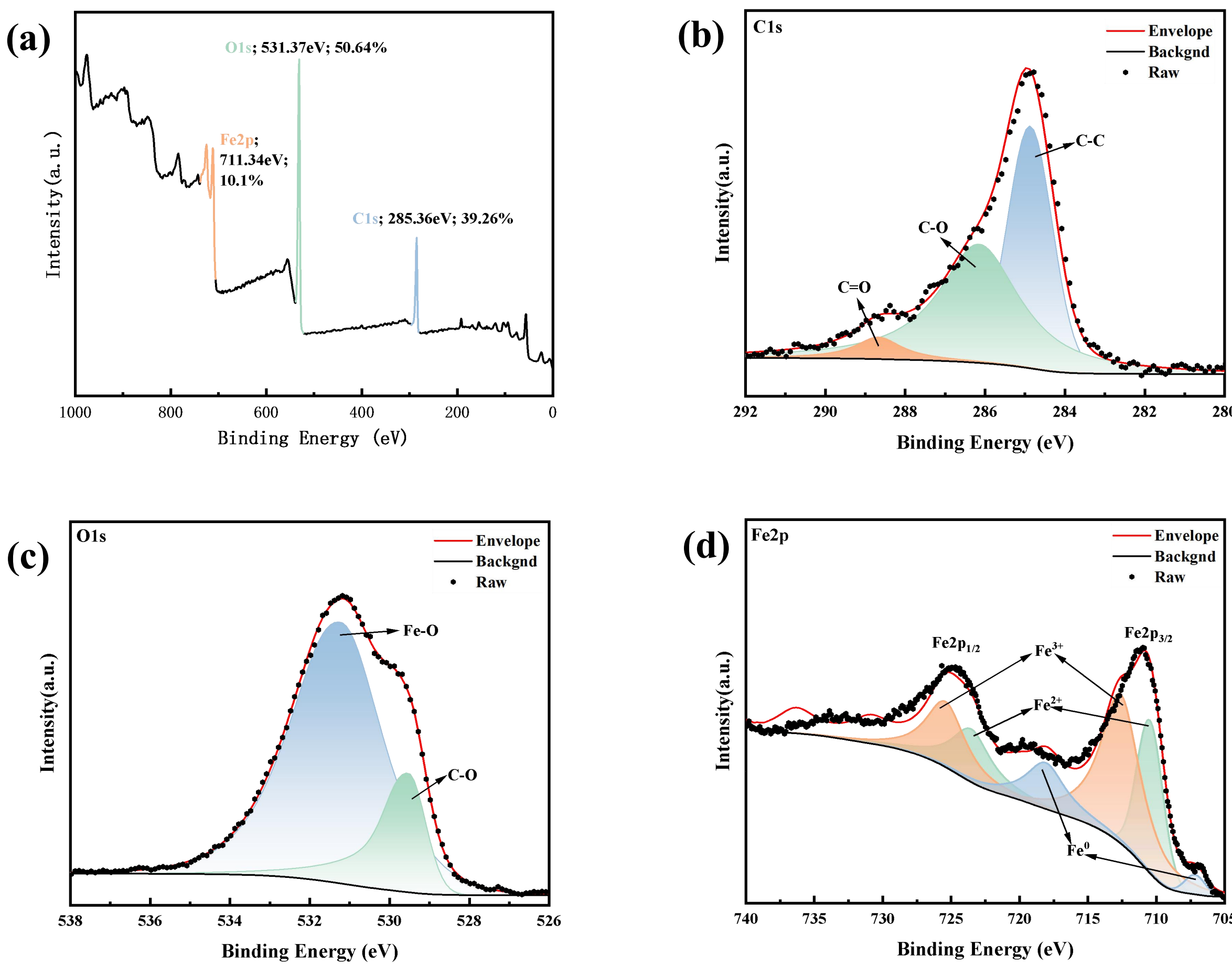

Fig S1. XPS spectrum of NZVI@HA: Full (a), C1s (b), O1s (c) and Fe2p (d) bound state levels

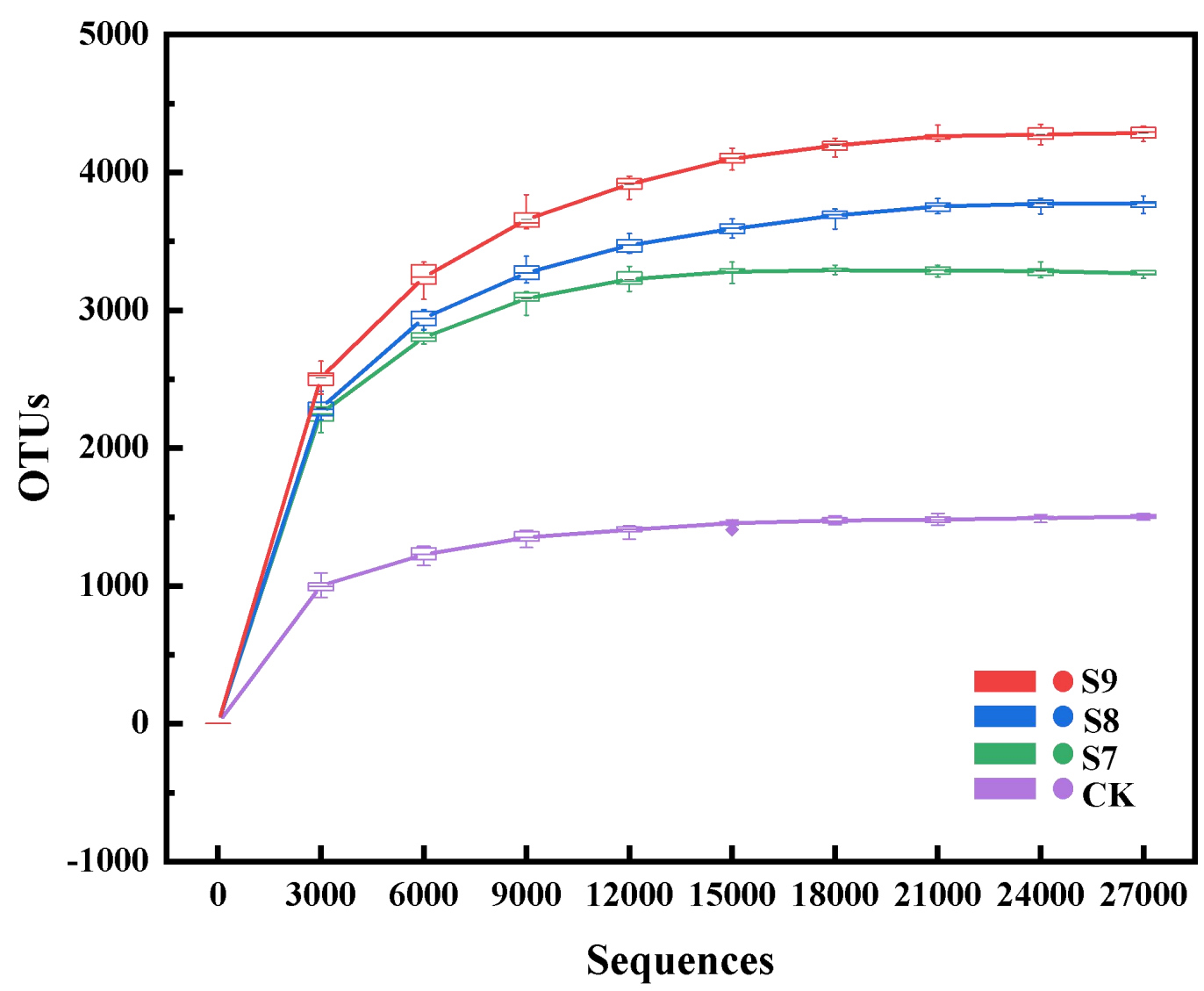

Fig S2. Rarefaction curve of bacterial community in soil samples

References

Chai, L., Ding, C., Tang, C., Yang, W., Yang, Z., Wang, Y., Liao, Q. & Li, J. 2018. Discerning three novel chromate reduce and transport genes of highly efficient Pannonibacter phragmitetus BB: From genome to gene and protein. *Ecotoxicology and Environmental Safety*.

Dai, M., Zhou, G., Ng, H. Y., Zhang, J., Wang, Y., Li, N., Qi, X., Miao, M., Liu, Q. & Kong, Q. 2019. Diversity evolution of functional bacteria and resistance genes (CzcA) in aerobic activated sludge under Cd(II) stress. *Journal of Environmental Management*.

Gu, R., Gao, J., Dong, L., Liu, Y., Li, X., Bai, Q., Jia, Y. & Xiao, H. 2020. Chromium metabolism characteristics of coexpression of ChrA and ChrT gene. *Ecotoxicology and Environmental Safety*.

Hussain, M. Z., Robertson, G. P., Basso, B. & Hamilton, S. K. 2020. Leaching losses of dissolved organic carbon and nitrogen from agricultural soils in the upper US Midwest. *Science of the Total Environment*.

Vilar, S., Gutierrez, A., Antezana, J., Carral, P. & Alvarez, A. 2005. A comparative study of three different methods for the sequential extraction of heavy metals in soil. *Toxicological & Environmental Chemistry*.
